# Supplementary material for: Discovery of the Clinical Candidate S‑892216: A Second-Generation of SARS-CoV‑2 3CL Protease Inhibitor for Treating COVID-19
Source: J Med Chem. 2025 Jul 5;68(20):21099–119. doi: 10.1021/acs.jmedchem.5c00754 (PMC12557365; doi:10.1021/acs.jmedchem.5c00754)
Supplement: Supplementary file 1 [file jm5c00754_si_001.pdf]

Supporting Information

Discovery of the Clinical Candidate S-892216: A Second-Generation of SARS-CoV-2 3CL Protease Inhibitor for Treating COVID-19

Yuto Unoh[1], Keiichiro Hirai[1], Shota Uehara[1], Sho Kawashima[1], Haruaki Nobori[1], Jun Sato[1], Hiromitsu Shibayama[1], Akihiro Hori[1], Kenji Nakahara[1], Kana Kurahashi[1], Masayuki Takamatsu[1], Shiho Yamamoto[1], Qianhui Zhang[1], Miki Tanimura[1], Reiko Dodo[1], Yuki Maruyama[1], Hirofumi Sawa[2,3,4,5], Ryosuke Watari[1], Tetsuya Miyano[1], Teruhisa Kato[1], Takafumi Sato[1], Yuki Tachibana[1]*

[1]Shionogi Pharmaceutical Research Center, 3-1-1 Futaba-cho, Toyonaka, Osaka 561-0825, Japan
[2]Institute for Vaccine Research and Development (HU-IVReD), Hokkaido University, Sapporo 001-0021, Japan
[3]International Institute for Zoonosis Control, Hokkaido University, Sapporo 001-0020, Japan
[4]One Health Research Center, Hokkaido University, Sapporo 060-0818, Japan
[5]Global Virus Network, 12901 Bruce B. Downs Blvd. MDC02, Tampa, Florida 33612, United States

*Corresponding author, E-mail: yuto.unoh@shionogi.co.jp (Yuto Unoh)

Table of contents

- Figure S1. Crystal structure of 3CL^{pro} in complex with compound **1** S3
- Figure S2. **S-892216** resistant SARS-CoV-2 isolation study S4
- Figure S3. Relationship between dose and C_{max} or $AUC_{0\text{-}48hr}$ of simulated **S-892216** plasma concentrations after repeated oral administration of **S-892216** twice daily in infected mice S5
- Table S1. Selectivity of **S-892216** against human and HIV protease S6
- Table S2. Antiviral activity of **S-892216** against various SARS-CoV-2 variants S6
- Table S3. A list of 3CL^{pro} residues that directly contact or are located within 5Å of **S-892216**..........S7
- Table S4. Transport assessment using human P-gp expressing LLC-PK1 cells monolayers................S8
- Table S5. Antiviral activity of **S-892216**, ensitrelvir, and nirmatrelvir against SARS-CoV-2 using VeroE6/TMPRSS2 cells with P-gp inhibitor and A549/ACE2-TMPRSS2 cells S8
- Table S6. Potency shift of antiviral activity of **S-892216** and ensitrelvir in the presence of human or mouse serum S8
- Table S7. Antiviral activity of **S-892216**, ensitrelvir, and nirmatrelvir against beta-coronavirus and alpha-coronavirus S9
- Table S8. Drug susceptibility of rgSARS-CoV-2 with 3CL^{pro} mutations associated with reduced susceptibility to ensitrelvir and nirmatrelvir S9
- Table S9. Antiviral activity of compounds against rgSARS-CoV-2 with 3CL^{pro} mutations associated with reduced susceptibility to **S-892216**, ensitrelvir, and nirmatrelvir..S10
- Table S10. Diffraction data and refinement statistics for SARS-CoV-2 3CL^{pro} complexed with compound **1**, **4** and **17** (**S-892216**) S11
- Experimental Procedures for *in vitro* pharmacological assays S12
- Synthesis of [^{14}C]-**S-892216** S14
- ^{1}H-NMR and ^{13}C-NMR spectra for synthesized compounds S18
- HPLC chromatogram of compound **17** (**S-892216**)..S36
- HPLC chromatogram of compound **18**...S37
- Physicochemical Properties of **17** (**S-892216**) S38
- HRMS Spectra for compound **17** (**S-892216**) S39
- References...S40

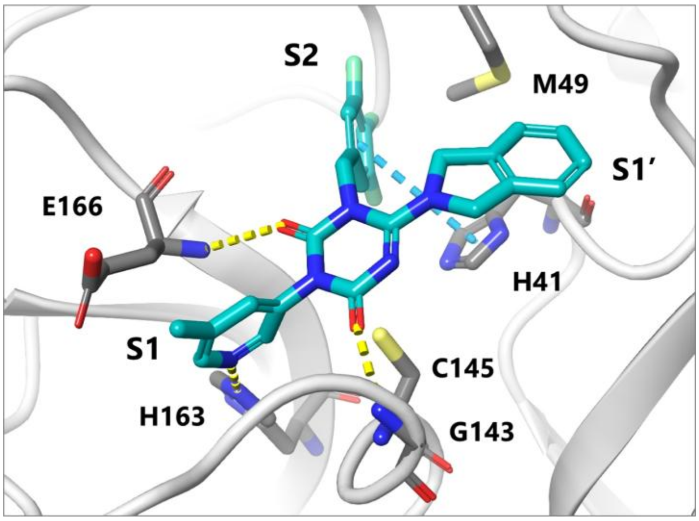

Figure S1. Crystal structure of $3CL^{pro}$ in complex with compound **1**. The two carbonyl groups interacted with G143 and E166. The 3-methylpyridine group in S1 pocket formed a hydrogen bond with H163.

A

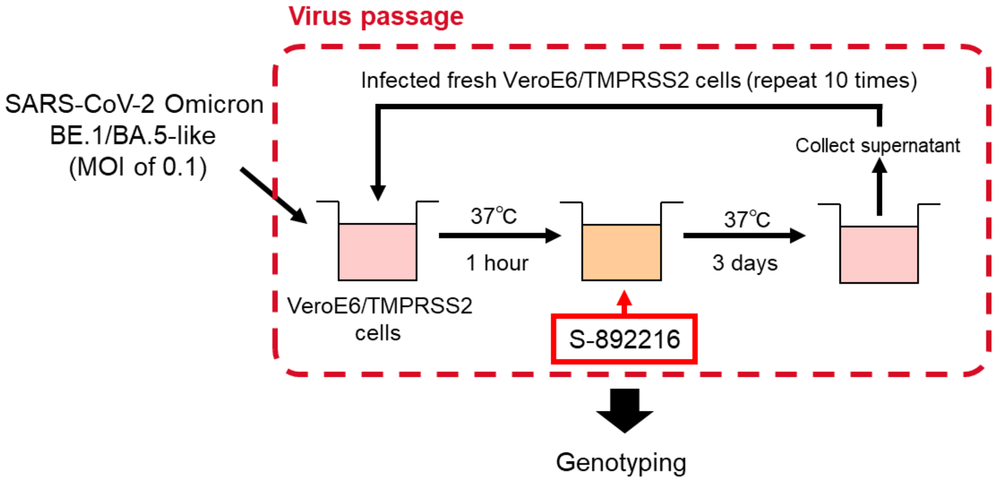

B

**S-892216** (nM)		No.	Amino acid substitutions
Passage 1-5	Passage 6-10		in 3CL^{pro} region
0	0	1	-
		2	-
		3	-
5.56	5.56	1	P252L
		2	L50F, P252L
		3	P252L
5.56	16.7	1	L50F, P252L
		2	L50F, P252L
		3	L50F, P252L
16.7	16.7	1	M49K, N221K, P252L
		2	M49K, P252L
		3	D48E, L50F, P252L
16.7	50.0	1	M49K, P252L
		2	M49K, P252L
		3	D48E, L50F, P252L
50.0	50.0	1	No virus
		2	No virus
		3	No virus

Figure S2. S-892216 resistant SARS-CoV-2 isolation study. (A) Passaging scheme for *in vitro* **S-892216** resistant isolation study. VeroE6/TMPRSS2 cells were infected with SARS-CoV-2 Omicron BE.1/BA.5-like strain and passaged to fresh VeroE6/TMPRSS2 cells every 3 days with **S-892216** for three independent wells. This subculture was repeated 10 times, and when CPE was observed at the fifth subculture, **S-892216** concentration was increased three-fold. (B) Mutations detected in 3CL^{pro} during the *in vitro* passage study using **S-892216**.

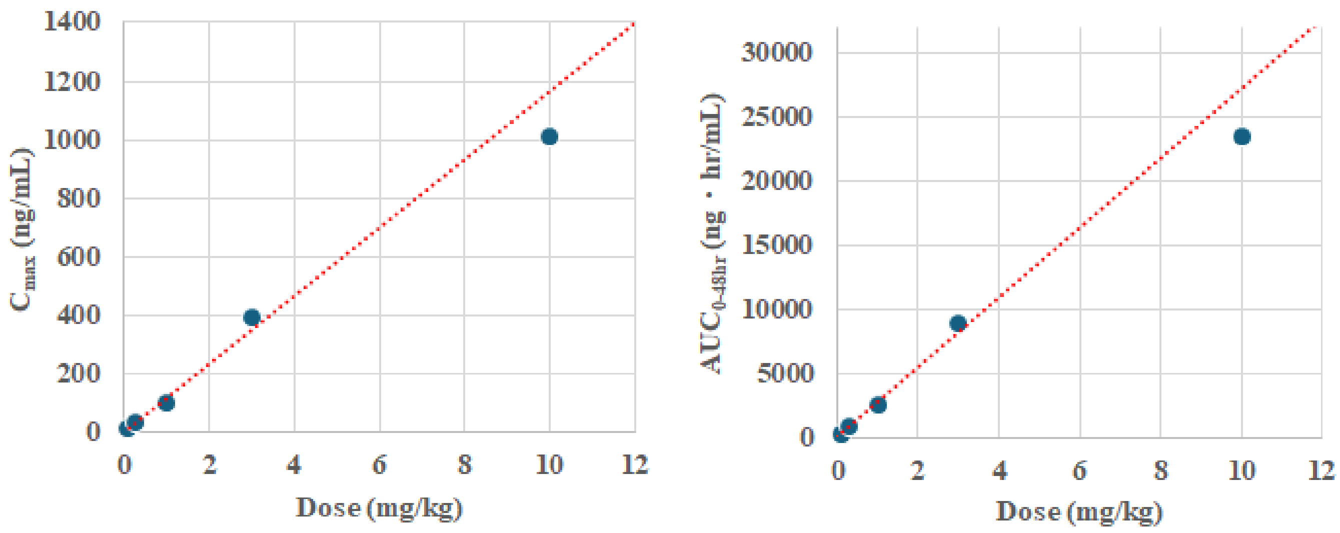

Figure S3. Relationship between dose and C_{max} or $AUC_{0\text{-}48hr}$ of simulated **S-892216** plasma concentrations after repeated oral administration of **S-892216** twice daily in infected mice. Simulated plasma concentrations in Figure 5D are used for the calculation of C_{max} and $AUC_{0\text{-}48hr}$.The red dotted line is an extrapolation from 0 to 0.1 mg/kg (minimum dosage).

Table S1. Selectivity of **S-892216** against human and HIV protease

Protease	IC_{50} (nM)
Human Caspase 2	>10,000
Human Chymotrypsin	>10,000
Human Cathepsin B/D/G/L	>10,000
Human Thrombin	>10,000
HIV-1 Protease	>10,000

Table S2. Antiviral activity of **S-892216** against various SARS-CoV-2 variants in a CPE inhibition assay using VeroE6/TMPRSS2 cells

SARS-CoV-2 strains	WHO Label	Pango Lineage	EC_{50} (nM)	
			**S-892216**	Ensitrelvir
hCoV-19/Japan/TY/WK-521/2020	Ancestral	A	8.77 ± 1.92	345 ± 102
hCoV-19/Japan/QHN002/2020	Alpha	B.1.1.7	3.85 ± 1.41	316 ± 38
hCoV-19/Japan/TY8-612/2021	Beta	B.1.351	9.64 ± 1.20	393 ± 109
hCoV-19/Japan/TY7-501/2021	Gamma	P.1	8.91 ± 1.62	622 ± 113
hCoV-19/Japan/TY11-927/2021	Delta	B.1.617.2	4.91 ± 1.29	338 ± 46
hCoV-19/Japan/TY38-871/2021	Omicron	BA.1.1	2.63 ± 0.18	136 ± 16
hCoV-19/Japan/TY40-385/2022	Omicron	BA.2	7.38 ± 3.82	257 ± 133
hCoV-19/Japan/TY41-721/2022	Omicron	BA.2.12.1	2.27 ± 0.26	198 ± 75
hCoV-19/Japan/TY41-716/2022	Omicron	BA.2.75	8.44 ± 1.25	217 ± 80
hCoV-19/Japan/TY41-703/2022	Omicron	BA.4.1	2.45 ± 0.40	178 ± 60
hCoV-19/Japan/TY41-763/2022	Omicron	BA.4.6	6.91 ± 1.21	302 ± 74
hCoV-19/Japan/TY41-704/2022	Omicron	BA.5.2.1	6.80 ± 1.38	366 ± 18
hCoV-19/Japan/TY41-702/2022	Omicron	BE.1/BA.5-like	6.54 ± 3.49	259 ± 66
hCoV-19/Japan/TY41-820/2022	Omicron	BF.7	9.49 ± 0.56	512 ± 74
hCoV-19/Japan/TY41-828/2022	Omicron	BF.7.4.1	7.39 ± 1.97	552 ± 81
hCoV-19/Japan/TY41-796/2022	Omicron	BQ.1.1	11.0 ± 1.7	531 ± 84
hCoV-19/Japan/TY41-832/2022	Omicron	CH.1.1.11	5.69 ± 2.44	375 ± 91
hCoV-19/Japan/TY41-795/2022	Omicron	XBB.1	5.11 ± 2.15	259 ± 41
hCoV-19/Japan/23-018/2022	Omicron	XBB.1.5.19	7.40 ± 1.08	570 ± 74
hCoV-19/Japan/TY41-951/2023	Omicron	XBB.1.9.1	12.5 ± 0.9	986 ± 111
hCoV-19/Japan/TY41-984/2023	Omicron	XBB.1.16	7.06 ± 2.00	331 ± 26
hCoV-19/Japan/TY41-831/2022	Omicron	XBF	6.27 ± 3.30	289 ± 15
hCoV-19/Japan/TY41-686/2022	Omicron	XE	7.10 ± 2.59	209 ± 88

Data are the means ± SD; n=3 biological replicates. Beta variant carries amino acid substitution of K90R and Omicron variants carry amino acid substitution of P132H in $3CL^{pro}$.

Table S3 A list of $3CL^{pro}$ residues that directly contact or are located within 5Å of **S-892216**

$3CL^{pro}$ residue within 5Å of **S-892216**	Interaction between $3CL^{pro}$ and **S-892216**
T25	-
T26	-
L27	-
H41	Edge to face π-π interaction between side chain
C44	-
T45	-
S46	-
M49	-
Y54	-
F140	-
L141	-
N142	-
G143	Hydrogen bond with main chain NH donor
S144	-
C145	Covalent bond with side chain S atom
H163	Hydrogen bond with side chain NH donor
H164	-
M165	-
E166	Hydrogen bond to main chain NH donor<br>CH-π interaction between side chain β-carbon
H172	-
V186	-
D187	-
R188	-
Q189	-

Table S4. Transport assessment using human P-gp expressing LLC-PK1 cells monolayers at 37°C for 120 minutes.

Compound	Concentration (μM)	Cell (LLC-PK1)	P_{app} ($\times 10^{-6}$ cm/sec)		Efflux ratio	Corrected efflux ratio
			Apical to Basolateral	Basolateral to Apical		
**S-892216**	0.5	P-gp	2.48	22.4	9.0	7.6
		Control	6.17	7.36	1.2	
Nirmatrelvir	0.5	P-gp	0.908	9.39	10.3	4.5
		Control	0.615	1.42	2.3	
Ensitrelvir[S1]	0.5	P-gp	2.03 ± 0.07	30.2 ± 1.8	14.9	13.3
		Control	10.3 ± 0.3	11.6 ± 0.3	1.1	

P_{app} value represents the mean of two samples (**S-892216** and nirmatrelvir) or the mean ± SD of four samples (ensitrelvir).

Table S5. Antiviral activity of **S-892216**, ensitrelvir, and nirmatrelvir against SARS-CoV-2 in a CPE inhibition assay using VeroE6/TMPRSS2 cells with or without P-gp inhibitor and A549/ACE2-TMPRSS2 cells

SARS-CoV-2	Cells		EC_{50} (nM) **S-892216**	Ensitrelvir	Nirmatrelvir
Ancestral, A	VeroE6/TMPRSS2 cells	without P-gp inhibitor	9.35 ± 1.04	285 ± 122	4880 ± 2030
		with P-gp inhibitor	3.36 ± 0.42	94.9 ± 38.6	68.1 ± 16.2
	A549/ACE2-TMPRSS2 cells		2.21 ± 0.63	73.1 ± 16.4	47.5 ± 12.3

Data are the means ± SD; n=3 biological replicates (n=12 biological replicates for **S-892216** in A549/ACE2-TMPRSS2 cells).

Table S6. Potency shift of antiviral activity of **S-892216** and ensitrelvir in the presence of human or mouse serum using VeroE6/TMPRSS2 cells

Compound	Serum	EC_{90} (nM) 0%	12.5%	25%	50%	100% (Extrapolated)	Potency shift (-fold)
**S-892216**	Human	1.66 ± 0.14	1.98 ± 0.08	2.76 ± 0.66	4.81 ± 0.88	7.90 ± 1.61	4.78 ± 1.05
	Mouse	1.66 ± 0.14	2.43 ± 0.32	3.42 ± 0.55	11.1 ±5.2	19.3 ± 9.3	11.7 ± 4.6
Ensitrelvir	Human	118 ± 52	367 ± 69	608 ± 75	838 ± 191	1590 ± 330	14.9 ± 6.1
	Mouse	118 ± 52	218 ± 60	462 ± 129	1560 ± 290	2920 ± 550	27.8 ± 10.9

Data are the means ± SD; n=3 biological replicates.

Table S7. Antiviral activity of **S-892216**, ensitrelvir, and nirmatrelvir against beta-coronavirus and alpha-coronavirus in a CPE inhibition assay using VeroE6/TMPRSS2 cells and MRC5 cells

			EC_{50} (nM)		
Cells	Virus		**S-892216**	Ensitrelvir	Nirmatrelvir
VeroE6/TMPRSS2 cells	Beta-coronavirus	SARS-CoV	6.61 ± 5.36	210 [S2]	NT
VeroE6/TMPRSS2 cells		MERS-CoV	57.3 ± 15.1	1400 [S2]	NT
MRC5 cells		HCoV-OC43	92.0 ± 21.0	142 ± 9	58.8 ± 12.2
MRC5 cells	Alpha-coronavirus	HCoV-229E	3570 ± 510	5500 [S2]	696 ± 210

Data are the means ± SD; n=3 biological replicates.

Table S8. Drug susceptibility of rgSARS-CoV-2 with $3CL^{pro}$ mutations associated with reduced susceptibility to ensitrelvir and nirmatrelvir

**rgSARS-CoV-2**	Fold change* v.s. wild-type			
**with $3CL^{pro}$ mutations**	**S-892216**	Ensitrelvir	Nirmatrelvir	Remdesivir
T25A	0.513	14.4 [S3]	1.06 [S3]	1.31 [S3]
M49I	0.137	5.25 [S3]	0.248 [S3]	0.731 [S3]
M49L	0.420	33.0 [S3]	0.833 [S3]	0.996 [S3]
S144A	0.697	8.24 [S3]	1.40 [S3]	1.49 [S3]
T21I+E166V	0.276	2.76 [S3]	36.0 [S3]	0.876 [S3]
L50F+E166V	0.405	2.81 [S3]	43.4 [S3]	0.672 [S3]

*Calculated by dividing the mean EC_{50} of each tested mutant by that of the cognate wild-type.

Table S9. Antiviral activity of compounds against rgSARS-CoV-2 with 3CL^{pro} mutations associated with reduced susceptibility to **S-892216**, ensitrelvir, and nirmatrelvir

	**rgSARS-CoV-2**	EC_{50} (nM)			
	**with 3CL^{pro} mutations**	**S-892216**	Ensitrelvir	Nirmatrelvir	Remdesivir
Assay 1	wild-type	6.70 ± 0.95	137 ± 30	3110 ± 510	1800 ± 190
	D48E	14.2 ± 2.3	171 ± 50	3350 ± 710	2010 ± 80
	M49K	24.5 ± 9.5	215 ± 97	1210 ± 220	1530 ± 140
	M49R	91.7 ± 19.8	300 ± 145	2550 ± 280	2590 ± 180
	N221K	11.8 ± 0.9	178 ± 56	3710 ± 720	1110 ± 120
	M49K+P252L	28.4 ± 10.7	200 ± 106	1420 ± 270	2450 ± 130
	L50F+P252L	21.8 ± 2.7	216 ± 74	6270 ± 1300	1410 ± 120
	D48E+L50F+P252L	39.0 ± 8.8	275 ± 103	7310 ± 1290	1510 ± 190
	M49K+N221K+P252L	34.7 ± 11.0	219 ± 67	1960 ± 351	2530 ± 80
Assay 2	wild-type	6.96 ± 1.51	119 ± 14	2970 ± 400	2010 ± 50
	T25A	3.57 ± 0.25	1710 ± 340	3150 ± 430	2630 ±260
	M49I	0.951 ± 0.046	625 ± 154	738 ± 134	1470 ± 110
	L50F	10.5 ± 0.7	140 ± 40	3970 ± 360	1560 ± 450
	P252L	8.49 ± 0.81	129 ± 20	3420 ± 350	1830 ± 320
	T21I+E166V	1.92 ± 0.49	328 ± 173	107000 ± 39900	1760 ± 120
	L50F+E166V	2.82 ± 0.63	334 ± 144	129000 ± 11800	1350 ± 160
Assay 3	wild-type	8.76 ± 0.53	165 ± 52	3780 ± 760	2380 ± 280
	M49L	3.68 ± 0.97	5450 ± 1760	3150 ± 1010	2370 ± 300
	S144A	6.11 ± 2.35	1360 ± 750	5290 ± 1370	3540 ± 530

Data are the means ± SD; n=3 biological replicates.

Table S10. Diffraction data and refinement statistics for SARS-CoV-2 3CL^{pro} complexed with compound **1**, **4** and **17** (**S-892216**)

	3CL^{pro} -Compound **1**	3CL^{pro} -Compound **4**	3CL^{pro}-Compound **17 (S-892216)**
**PDB code**	9LVR	9LVT	9LVV
**Data collection**			
Space group	P212121	P21	I2
Cell dimensions			
a, b, c (Å)	67.83 101.25 104.18	56.01 99.05 58.76	44.46 53.73 114.31
α, β, γ (°)	90.00 90.00 90.00	90.00 108.16 90.00	90.00 98.88 90.00
Wavelength (Å)	1.54178	1.54178	1.54178
Resolution (Å)	30.22-2.20 (2.27-2.20)	28.42-1.90(1.94–1.90)	27.70-1.90(1.95-1.90)
Completeness (%)	99.9(100.0)	99.6(99.1)	99.6(97.8)
R_{merge} (%) *	17.1(51.3)	5.1(33.8)	3.9(18.4)
$I/\sigma(I)$	9.1(3.7)	21.3(3.6)	18.3(5.2)
**Refinement**			
Resolution (Å)	30.23 – 2.20	28.44 - 1.90	27.72 - 1.90
No. of reflections	35092	45336	19970
R_{work}/R_{free}	0.2226/0.2684	0.1952/0.2371	0.1912/0.2417
B factor (Å^2)			
Protein	26.4	18.9	18.0
Ligand	38.9	17.3	18.7
Water	21.2	22.0	21.5
RMS deviations			
Bond length (Å)	0.0089	0.0073	0.0085
Bond angles (°)	1.6676	1.6470	1.7725
Ramachandran plot (%)			
Favored	94.4	96.9	95.2
Allowed	4.9	2.6	4.1
Outliers	0.7	0.5	0.7

Values in parentheses are for the highest resolution shell.

* $R_{merge} = \Sigma| I - \langle I \rangle |/\Sigma I$, where *I* is the observed intensity and $\langle I \rangle$ is the average intensity.

Human and HIV Protease Enzyme Assay

Selectivity tests against a variety of human and HIV protease activity were conducted by Eurofins Panlabs Discovery Services Taiwan, Ltd., on behalf of Shionogi Co. & Ltd. as per established protocols. **S-892216** was tested on a set of seven human proteases (caspase-2, chymotrypsin, cathepsin B/D/G/L, and thrombin) and HIV-1 protease at 10 μM.

Cellular Antiviral Activity using VeroE6/TMPRSS2 cells with P-gp inhibitor

Antiviral activity of compounds against SARS-CoV-2 in VeroE6/TMPSS2 cells with P-gp inhibitor was assessed by same method as **Cellular Antiviral Activity using VeroE6/TMPRSS2 cells**. 0.75 μmol/L CP-100356 monohydrochloride (Sigma-Aldrich) was added as a P-gp inhibitor. Cells were infected with SARS-CoV-2 ancestral strain (hCoV-19/Japan/TY/WK-521/2020) at 100 $TCID_{50}$/well and incubated for 3 days.

Cellular Antiviral Activity using A549-Dual hACE2-TMPRSS2 cells

Antiviral activity of compounds against SARS-CoV-2 in A549-Dual hACE2-TMPRSS2 cells was assessed by monitoring the cell viability. A549-Dual hACE2-TMPRSS2 cells (1.5×10^4 cells/well) suspended in MEM supplemented with 2% FBS and 1% P/S were seeded into 96-well plates with diluted compounds in each well. Cells were infected with SARS-CoV-2 Delta strain (hCoV-19/Japan/TY11-927/2021) at 100 $TCID_{50}$/well and cultured at 37 °C with 5% CO_2 for 2 days. Cell viability was assessed using a CellTiter-Glo 2.0 assay (Promega). EC_{50} values were determined by plotting the compound concentration versus inhibition and fitting data with a four-parameter logistical fit (Model 205, XLfit). The mean and SD values were calculated based on three (twelve for **S-892216**) independent experiments.

Cellular Antiviral Activity in the Presence of Human and Mouse Serum

The inhibitory effect of **S-892216** on SARS-CoV-2 replication in cultured cells in the presence of human and mouse serum was evaluated using viral replication inhibition assays as previously reported.[S4] Briefly, VeroE6/TMPRSS2 cells (1.5×10^4 cells/well) were seeded on 96-well plates in MEM supplemental with 2% FBS and 1% P/S 1 day prior to infection. After the removal of supernatant, the cells were infected with 1000 $TCID_{50}$ SARS-CoV-2 Gamma strain (hCoV-19/Japan/TY7-501/2021) and incubated for about 1 hour at 37°C, 5% CO_2. The supernatant including unadsorbed viruses were removed, and then added fresh medium with diluted compound and human serum (Sigma) or mouse serum (BALB/cAJcl mice, CLEA Japan, Inc.) (final 12.5, 25, or 50%). The plates were incubated for 1 day at 37°C, 5% CO_2, followed by extraction of the viral RNA from the cell lysate using TRIzol LS (Thermo Fisher Scientific Inc.) and a Direct-zol-96 RNA Kit (Zymo Research, Irvine, CA, USA). The viral RNA was quantified by real-time PCR using EXPRESS One-Step SuperScript qRT-PCR kit (Thermo Fisher Scientific Inc.) on an Applied Biosystems QuantStudio 5 system (Thermo Fisher Scientific) with SARS-CoV-2-specific primers and probes.[S5] EC_{90} values in the presence of each concentration of serum were calculated with two points sandwiching one-tenth reduction. EC_{90} values extrapolated to 100% serum were calculated by linear regression using the EC_{90} values of each serum concentration. Potency shifts were calculated by dividing EC_{90} extrapolated to 100% serum by EC_{90} in the absence of human and mouse serum. The mean and SD values were calculated based on three independent experiments.

Cellular Antiviral Activity Against Coronaviruses

Antiviral activity of compounds against SARS-CoV, MERS-CoV, HCoV-OC43, and human coronavirus 229E (HCoV-229E) was assessed by monitoring the cell viability. Antiviral activities against SARS-CoV and MERS-CoV were evaluated at Hokkaido University using VeroE6/TMPRSS2 cells as previously reported.[S2] Briefly, VeroE6/TMPRSS2 cells (1.5×10^4 cells/well) suspended in MEM supplemented with 2% FBS were seeded into 96-well plates with diluted compounds in each well. Cells were infected with each SARS-CoV at 1000 $TCID_{50}$/well or MERS-CoV 2500 $TCID_{50}$/well and cultured at 37 °C with 5% CO_2 for 3 days. Cell viability was assessed via (3-[4,5-dimethyl-2-thiazolyl]-2,5-diphenyl-2H-tetrazolium bromide (MTT) assay (Nacalai Tesque, Japan). Antiviral activity against HCoV-OC43 and HCoV-229E was evaluated using MRC-5 cells. MRC-5 cells (2.0×10^4 cells/well) suspended in MEM supplemented with 2% FBS and 1% P/S were seeded into 96-well plates and incubated at 37 °C with 5% CO_2 overnight. The next day, the cells were infected with HCoV-OC43 or HCoV-229E at 300 or 1,000 $TCID_{50}$/well, respectively, and incubated at 37 °C with 5% CO_2 for 1 h, followed by removal of the inoculum and addition of MEM supplemented with 2% FBS and 1% P/S with the diluted compounds. The plates were incubated at 37 °C with 5% CO_2 for 3 days. Cell viability was assessed using a CellTiter-Glo 2.0 assay and EC_{50} values were determined by plotting the compound concentration versus inhibition and fitting data with a four-parameter logistical fit (Model 205, XLfit). The mean and SD values were calculated based on three independent experiments.

Synthesis of [^{14}C]-S-892216

Synthesis of **[^{14}C]-S-892216** via an alternative ring synthesis approach is summarised in Scheme S1. For the detail discussion of **S-892216** synthesis routes, see references[S6,S7].

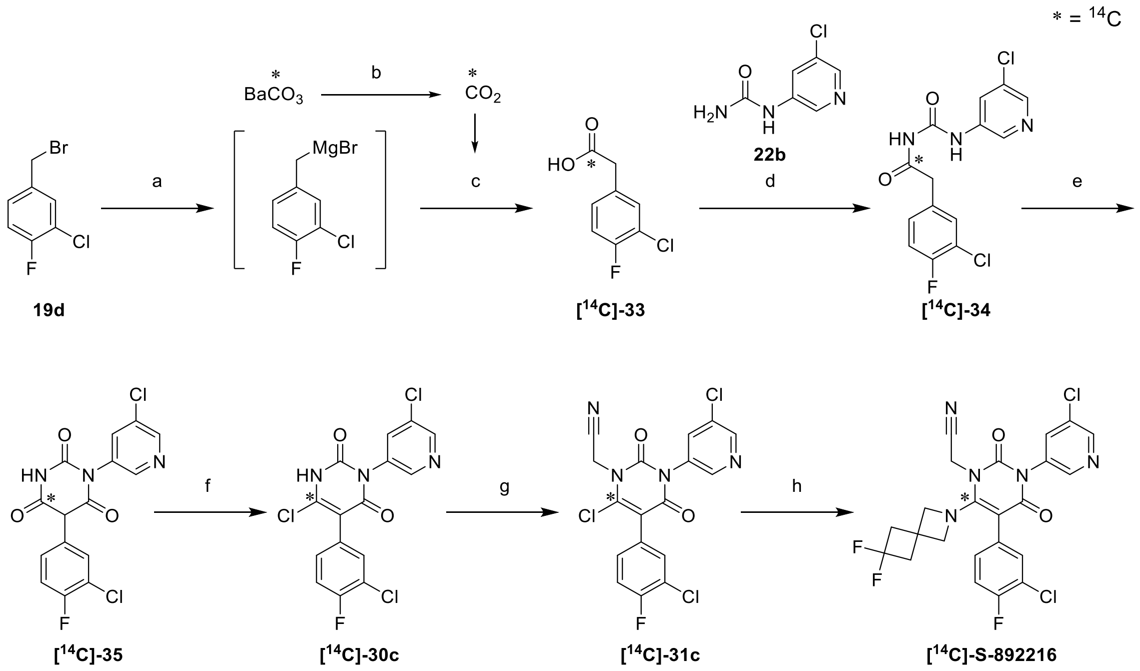

Scheme S1. Synthesis of **[^{14}C]-S-892216**.

(a) Mg, I_2, Et_2O, 35°C; (b) $HClO_4$; (c) -196°C to -78°C to rt; (d) **22b**, T_3P, Et_3N, DMA, rt; (e) CDI, DBU, THF, rt; (f) $POCl_3$, H_2O. 90°C; (g) 2-bromoacetonitrile, DIPEA, DMF, 40°C; (h) 6,6-difluoro-2-azaspiro[3.3]heptane trifluoroacetic acid salt, DIPEA, DMF, 60°C

2-(3-Chloro-4-fluorophenyl)acetic-1-^{14}C acid ([^{14}C]-33)

To a stirring suspension of Mg (55 mg, 1.98 mmol) in Et_2O (2 mL) was added a piece of I_2 and dropwise **19d** (418 mg, 1.98 mmol) in Et_2O (1 mL) and rinse out with Et_2O (1 mL) at r.t.. The resulting suspension was stirred at 35°C for 1.5 hours. The resulting mixture was cooled with liquid nitrogen and introduced [^{14}C]-carbon dioxide generated from barium [^{14}C]-carbonate (299 mg, Total activity (TA) = 86.3 mCi, Specific activity (SA) = 56.8 mCi/mmol, 1.52 mmol) and 60% perchloric acid by vacuum line. After stirring at about -70°C for 10 min, the reaction mixture was warmed slowly to 0°C for 2 hours to complete the reaction. The reaction mixture was quenched with water (2 mL) at 0°C and acidified (pH = 1) with 2M HCl aq. (2.5 mL) and extracted with Et_2O. The organic layer was extracted with 1M $NaHCO_3$ aq. (6 mL). The aqueous layer was acidified (pH = 2) with 2M HCl aq. (4 mL) and extracted with Et_2O. The organic layer was washed with brine, dried over sodium sulfate, filtered and evaporated under reduced pressure. The crude **[^{14}C]-33** (278 mg, 1.46 mmol, as white crystals) was used in next reaction without further purification. HPLC purity 92%

2-(3-Chloro-4-fluorophenyl)-*N*-((5-chloropyridin-3-yl)carbamoyl)acetamide-1-^{14}C ([^{14}C]-34)

To a stirring suspension of **[^{14}C]-33** (Theoretical value: 286 mg, 1.52 mmol) and **22b** (286 mg, 1.67 mmol) in DMA (2 mL) was added NEt_3 (0.242 mL, 1.74 mmol). After 10 minutes, the mixture was clear solution. To the reaction mixture was added T_3P (50% in AcOEt, 1.4 mL, 2.35 mmol) and stirred at 50°C for 2 hours. To the reaction mixture was added T_3P (total 4 mL) every hour. To the reaction mixture was added NEt_3 (total 1.3 mL) every 30 minutes and stirred at 50°C for 2 hours. The reaction mixture was evaporated under reduced pressure to remove AcOEt. Then the reaction mixture was cooled 0°C, H_2O (3.8 mL) was added and stirred at 0°C for 30 minutes. The precipitated solid were collected by filtration and washed with 50% $MeCN/H_2O$ (3 mL) to obtain **[^{14}C]-34** (432 mg, 1.26 mmol). HPLC purity 92%

5-(3-Chloro-4-fluorophenyl)-1-(5-chloropyridin-3-yl)pyrimidine-2,4,6(1*H*,3*H*,5*H*)-trione-4-^{14}C ([^{14}C]-35)

To a stirring suspension of **[^{14}C]-34** (432 mg, 1.26 mmol) in 2-Me-THF (4 mL) was added 1,1'-carbonyldiimidazole (328 mg, 2.02 mmol) and dropwise 1,8-diazabicyclo[5.4.0]-7-undecene (264 µL, 1.77 mmol) for 2 minutes. To a reaction mixture was added 2-Me-THF (1 mL) and stirred at r.t. for 1 h. The reaction mixture was poured into ice (ca 2 g), water 2 mL and 2M HCl aq. (4.5 mL), and rinsed out with 2-Me-THF (5 mL). The organic layer was separated. The aqueous layer was re-extracted twice with 2-Me-THF (10 mL). The organic layers were combined and evaporated in reduced pressure to ca. 2.5g. To a stirring solution of the residue was added toluene (7 mL) and acetic acid, and cooled under ice-bath. The suspension was stirred at 0°C for 1 h. The precipitated solid were collected by filtration and washed with cold toluene (1.86 mL) to obtain **[^{14}C]-35** (381 mg, 1.04 mmol) as yellow solid. Mother liquor was purified by column chromatography ($CHCl_3$/MeOH : 19/1 to 3/2) to obtain **[^{14}C]-35** (60 mg, 0.164 mmol) as yellow solid. HPLC purity 99%

6-Chloro-5-(3-chloro-4-fluorophenyl)-3-(5-chloropyridin-3-yl)pyrimidine-2,4(1*H*,3*H*)-dione-6-^{14}C ([^{14}C]-30c)

To a combined starting material **[^{14}C]-35** (441 mg, 1.20 mmol) was added $POCl_3$ (3 mL) at r.t. and stirred at 50°C for 10 minutes. To a reaction mixture (yellow slurry) was added water (65 µL) and stirred at 90°C for 12 hours and allowed to stand overnight at r.t.. The reaction mixture was stirred again at 90°C for 12 hours 40 minutes and allowed to stand overnight at r.t. An ice-cooled mixture of 30% KOHaq. (120 mmol) and THF (15 mL) was added dropwise to reaction mixture over 1 h under ice bath cooling. To the reaction mixture was added THF (10 mL) and toluene (12.5 mL) and stirred at r.t. for 15 minutes and rinse out with THF:toluene=2:1 (10 mL) and water (3 mL). The organic layer was separated. The aqueous layer was re-extracted twice with THF:toluene=2:1 (45 mL). The organic layers were washed with 20% KCl aq. (35 mL), combined and evaporated in reduced pressure. To a stirring solution of the residue in DMA (7 mL) was added acetic acid (510 µL) and warmed at 50°C. To a solution was added dropwise water (4.64 mL) over 2 minutes under stirring. After 13 minutes, the oil bath was removed. The suspension was stirred at r.t for 20 minutes and 0°C for 1 h. The precipitated crystals were collected and washed with 70% DMA aq. (2 mL) and water (5 mL) to give **[^{14}C]-30c** (345.3 mg, 0.893 mmol, as white crystals). To the mother liquor was added water (2 mL) under ice bath cooling. The precipitated crystals were collected and washed with 70% DMA aq. (1 mL) and water (5 mL) to give **[^{14}C]-30c** (57.3 mg, 0.148 mmol, as brown crystals). HPLC purity 97%.

2-(6-Chloro-5-(3-chloro-4-fluorophenyl)-3-(5-chloropyridin-3-yl)-2,4-dioxo-3,4-dihydropyrimidin-1(2*H*)-yl-6-^{14}C)acetonitrile ([^{14}C]-31c)

To a stirring solution of **[^{14}C]-30c** (402.6 mg, 1.036 mmol) in DMF (4 mL) was added 2-bromoacetonitrile (207 µL, 3.11 mmol) and DIPEA (543 µL, 3.11 mmol). The resulting mixture was stirred at 40°C for 3 h. To a reaction mixture was added EtOAc, 2N-HCl and water (4 mL) under ice bath cooling. The organic layer was separated and washed twice with water (4 mL). The aqueous layer was re-extracted with EtOAc. The organic layers were combined and washed with brine, dried over sodium sulfate, filtered and evaporated under reduced pressure. The residue was purified by silica gel column chromatography (*n*-hexane/EtOAc = 4/1 to 2/1) to give crude product. The crude product was re-purified by amino silica gel column chromatography (*n*-hexane/EtOAc = 1/1) followed by trituation and filtlation in IPE to give **[^{14}C]-31c** (366.8 mg, 0.858 mmol, as pale beige powder). HPLC purity 98%.

[^{14}C]-S-892216

To a stirring solution of **[^{14}C]-31c** (366 mg, 0.856 mmol) in DMF (3.7 mL) was added 6,6-difluoro-2-azaspiro[3.3]heptane 2,2,2-trifluoroacetate (254 mg, 1.027 mmol) and DIPEA (179 µL, 1.025 mmol), and stirred at 60°C. After 2 hours 15 minutes, to a reaction mixture was added azaspiro[3.3]heptane 2,2,2-trifluoroacetate (66.5 mg, 0.269 mmol) and DIPEA (47 µL, 0.257 mmol) and stirred at 60°C for 2 h. To a reaction mixture was added DIPEA (74 µL, 0.428 mmol) and stirred at 60°C for 1.5 h. After cooling to r.t., EtOAc (12 mL) and water (12 mL) were added. The organic layer was separated and washed twice with water. The aqueous layer was re-extracted with EtOAc. The organic layers were combined and washed with brine, dried over sodium sulfate, filtered and evaporated under reduced pressure. The crude product was re-purified by amino silica gel column chromatography (EtOAc only). The obtained eluate was roughly evaporated and crystallized. To an obtained suspension was added dropwise IPE (1.5 mL). The supernatant was removed by decanting. The residue was triturated with IPE (1 mL) and the supernatant was removed by decanting. The residue was dried under reduced pressure to give **[^{14}C]-S-892216** (412 mg, 0.673 mmol). HPLC purity 98.7%

Analytical HPLC was performed on a YMC-Triart (C_{18}, 5 µm, 3.0 × 150 mm, mobile phase [A] = 0.1% $HCOONH_4$ / H_2O, [B] = 0.1% $HCOONH_4$ / MeCN, flow rate 0.4 mL/min, at 40°C) using a Shimadzu HPLC system equipped with a Radiomatic 625TR (PerkinElmer Inc.), Cocktail: ULTIMA-FLO AP, flow rate 1 mL/min.

Time-program for gradient elution

Time (min)	Mobile phase B (%)
0	50
20	85
21	85
21.01	50
25	50

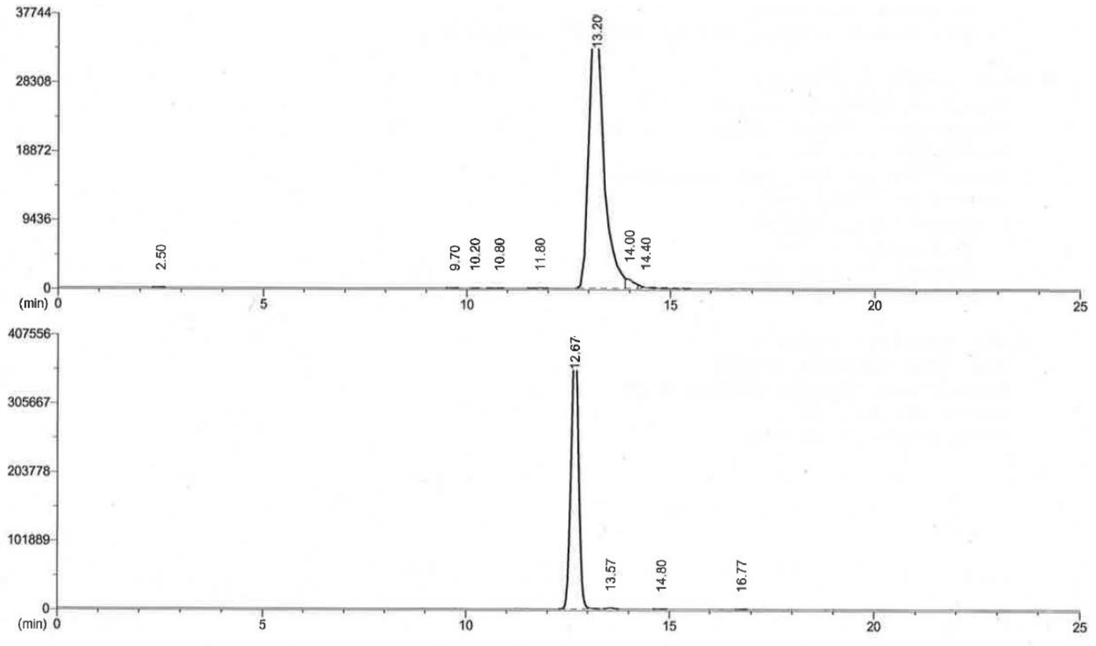

Channel 1: ^{14}C CPM

peak#	Rt (min)	Area	% Pks
1	2.50	19	0.01
2	9.70	9	0.01
3	10.20	9	0.01
4	10.80	28	0.02
5	11.80	56	0.03
6	13.20	162843	99.83
7	14.00	154	0.09
8	14.40	0	0
Total peak area		161118	

Channel 2: UV

peak#	Rt (min)	Area	% Pks
1	12.67	5.25E+06	99.72
2	13.57	12910	0.25
3	14.80	1040	0.02
4	16.77	878	0.02
Total peak area		5262702	

^{1}H NMR and ^{13}C NMR spectra for synthesized compounds

^{1}H NMR spectra of **1** in $CDCl_3$

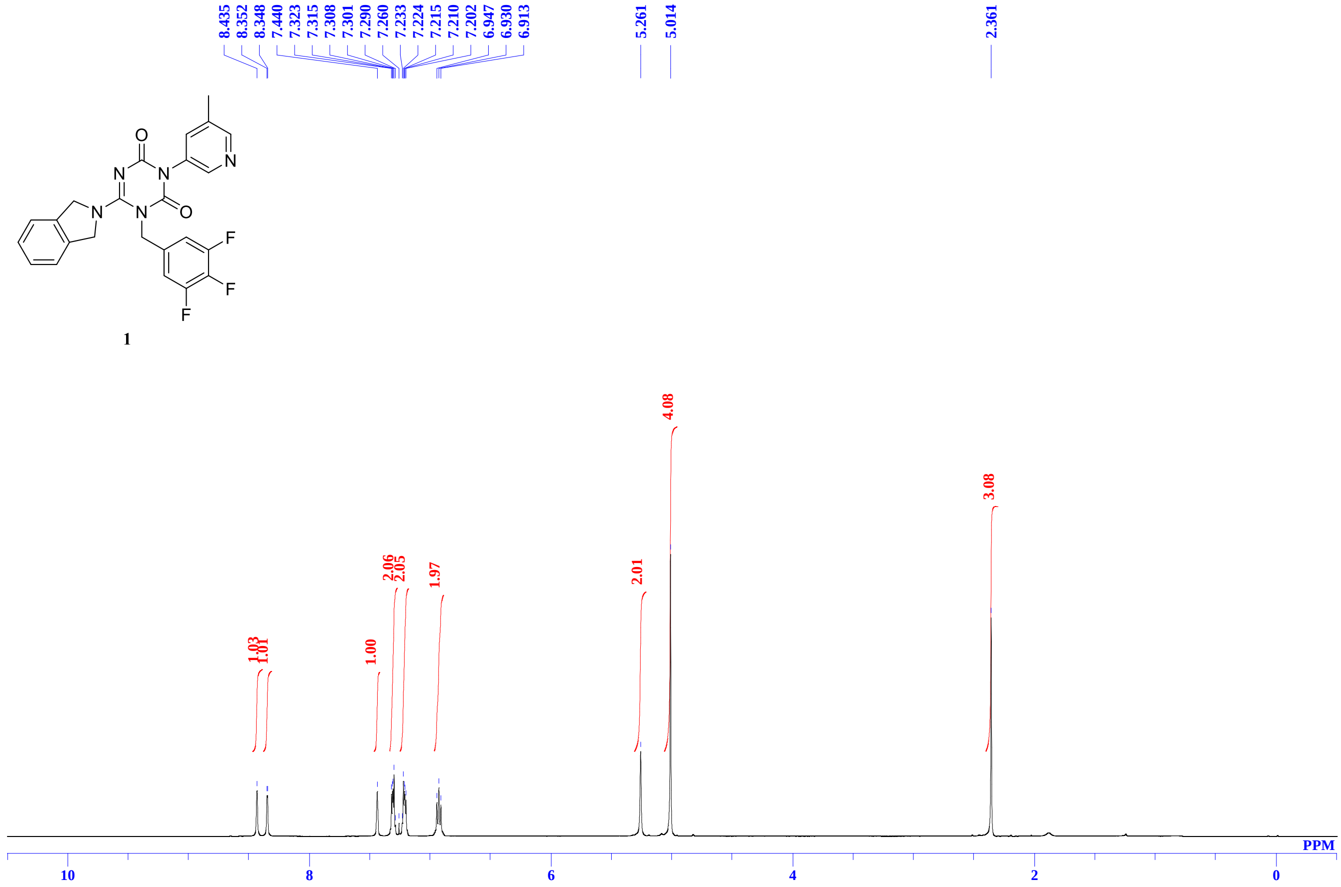

^{13}C NMR spectra of **1** in $CDCl_3$

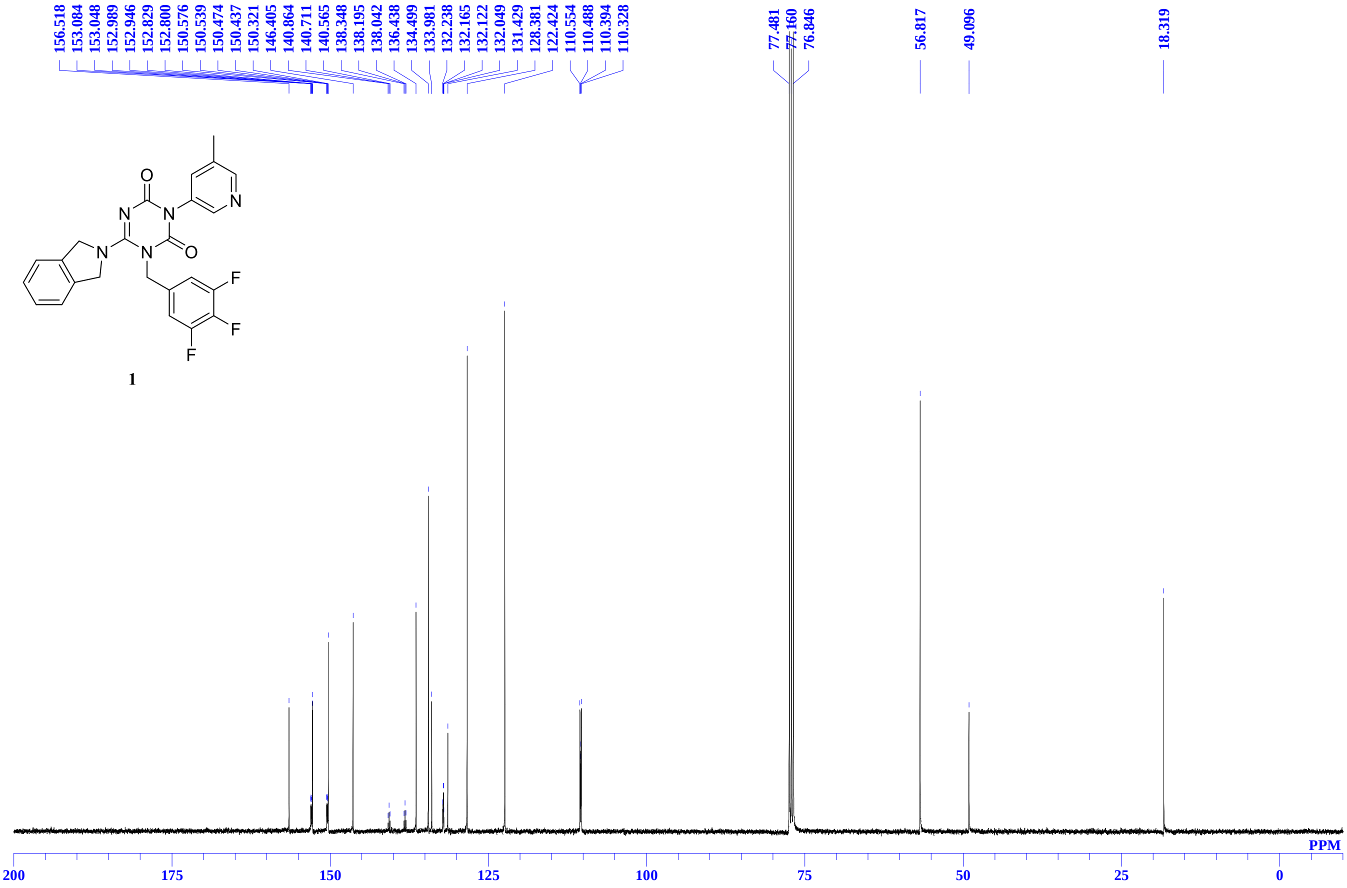

^{1}H NMR spectra of **2** in DMSO-d_6

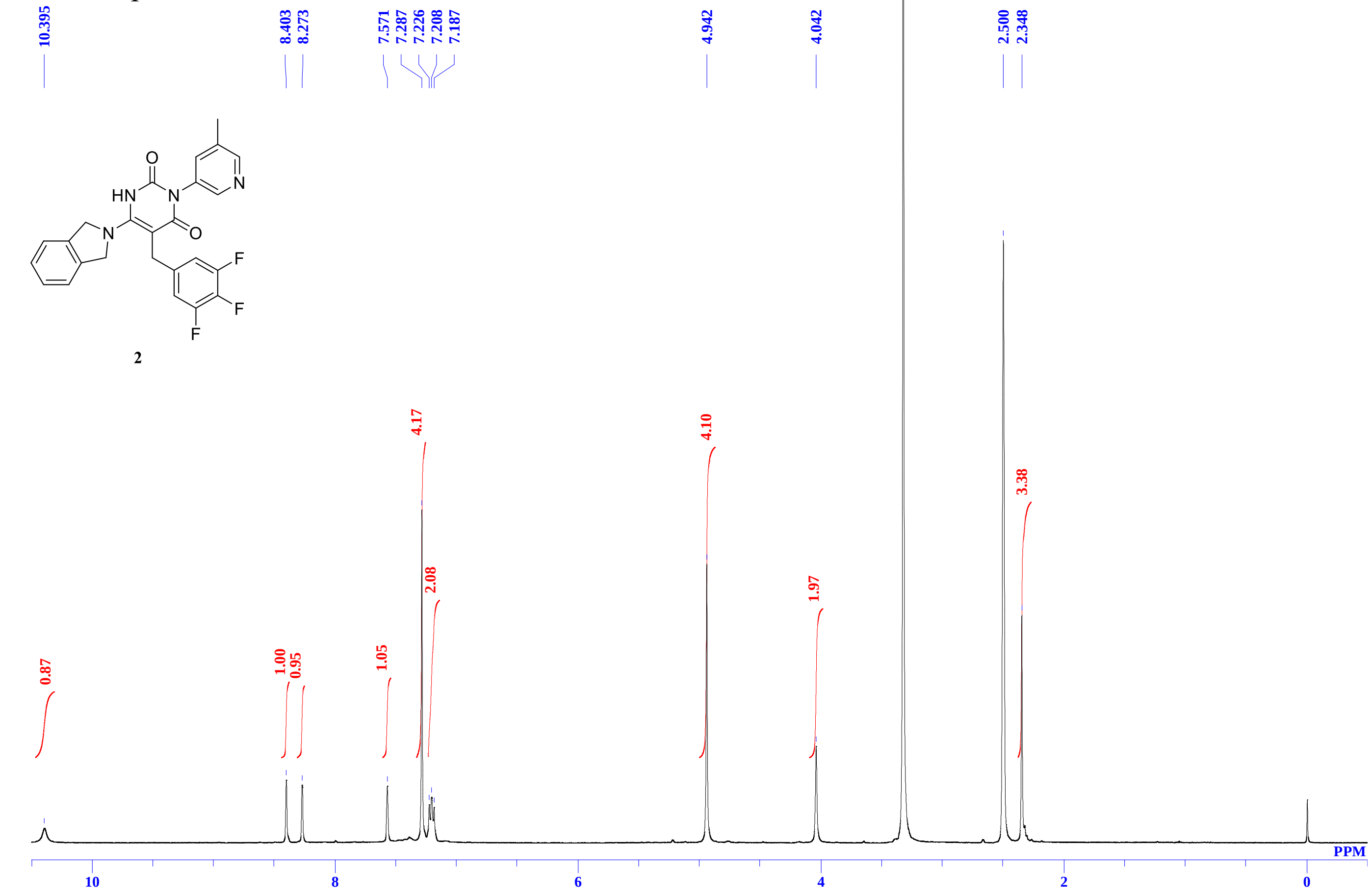

^{13}C NMR spectra of **2** in DMSO-d_6

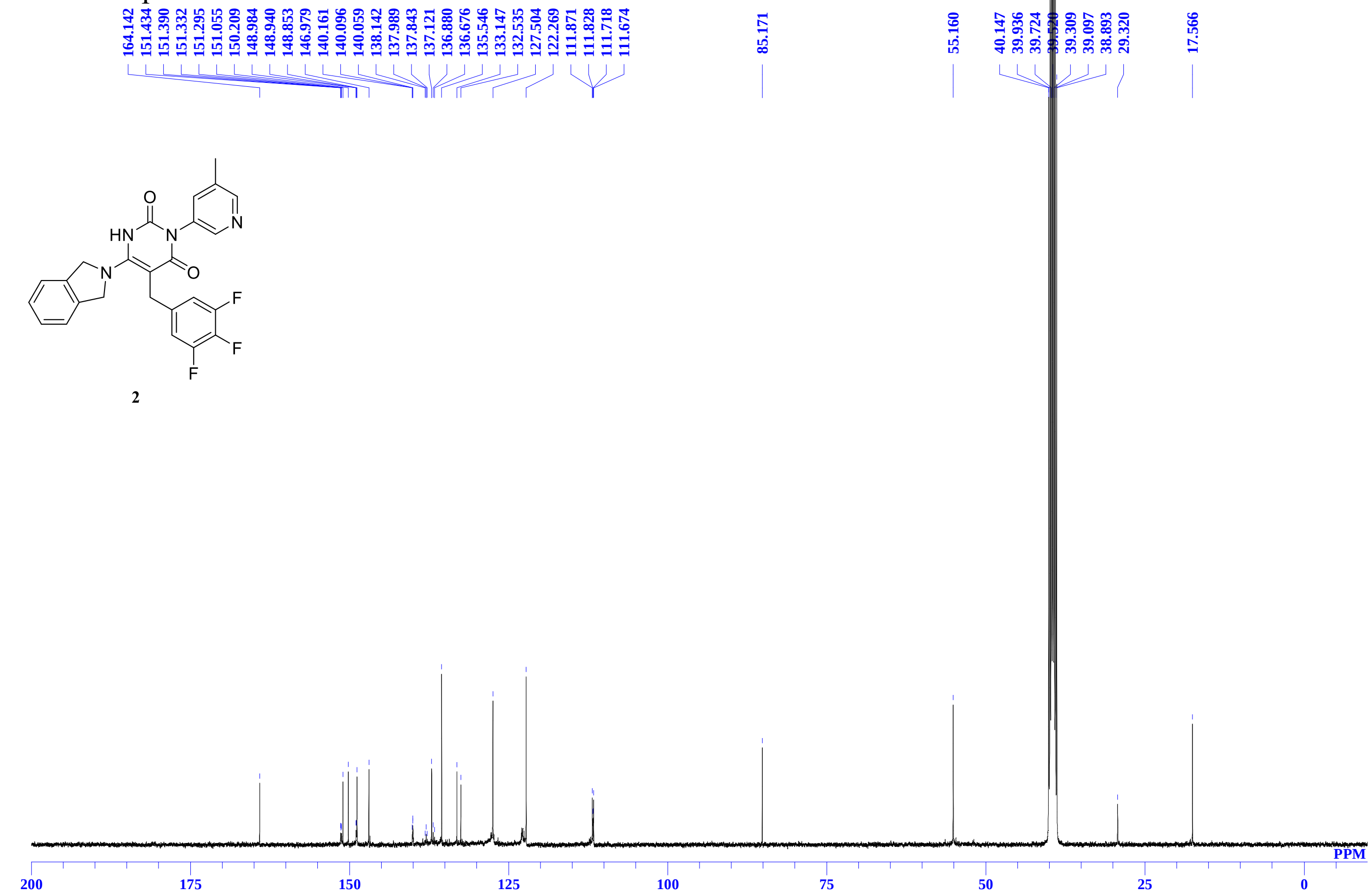

^{1}H NMR spectra of **3** in $CDCl_3$

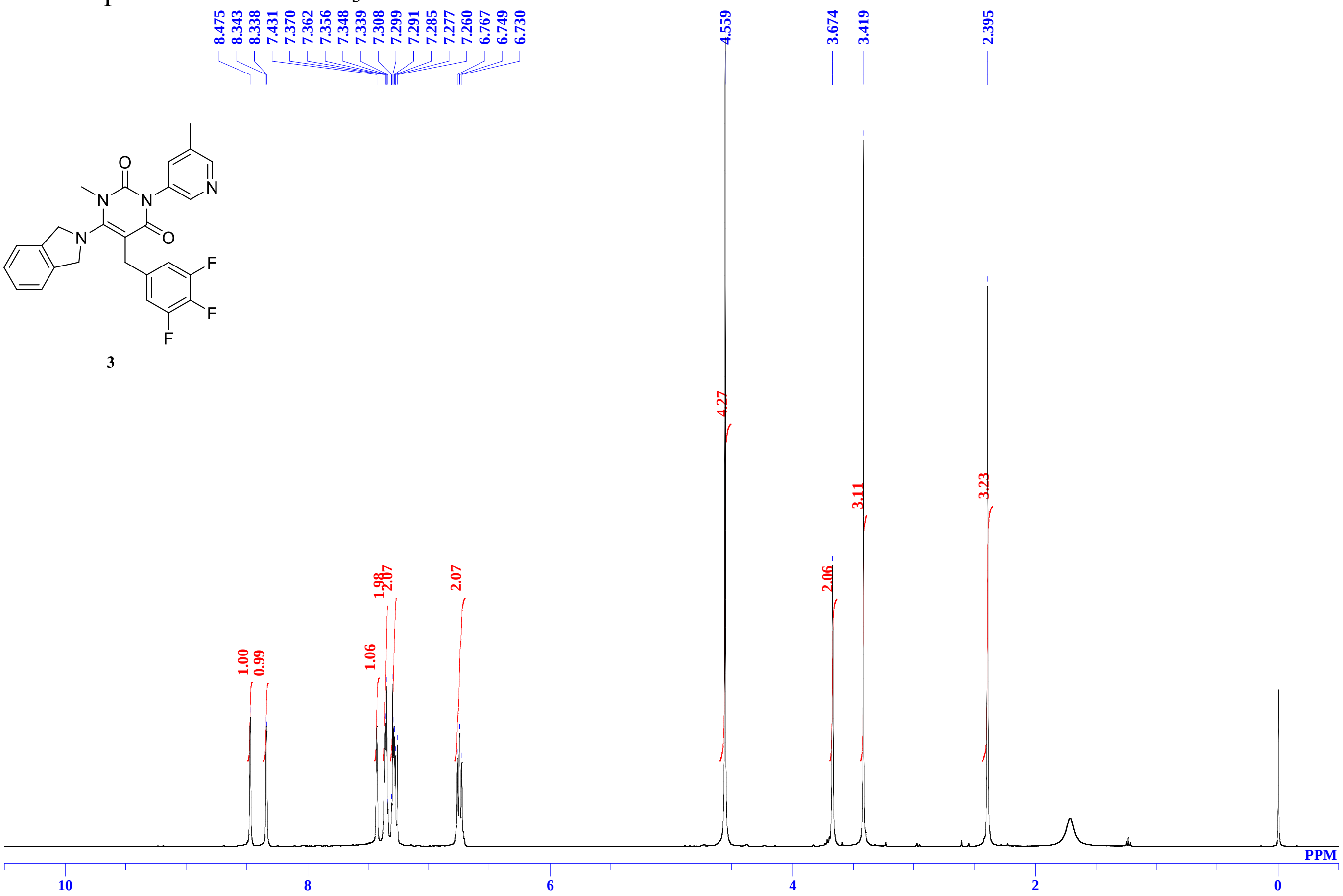

^{13}C NMR spectra of **3** in $CDCl_3$

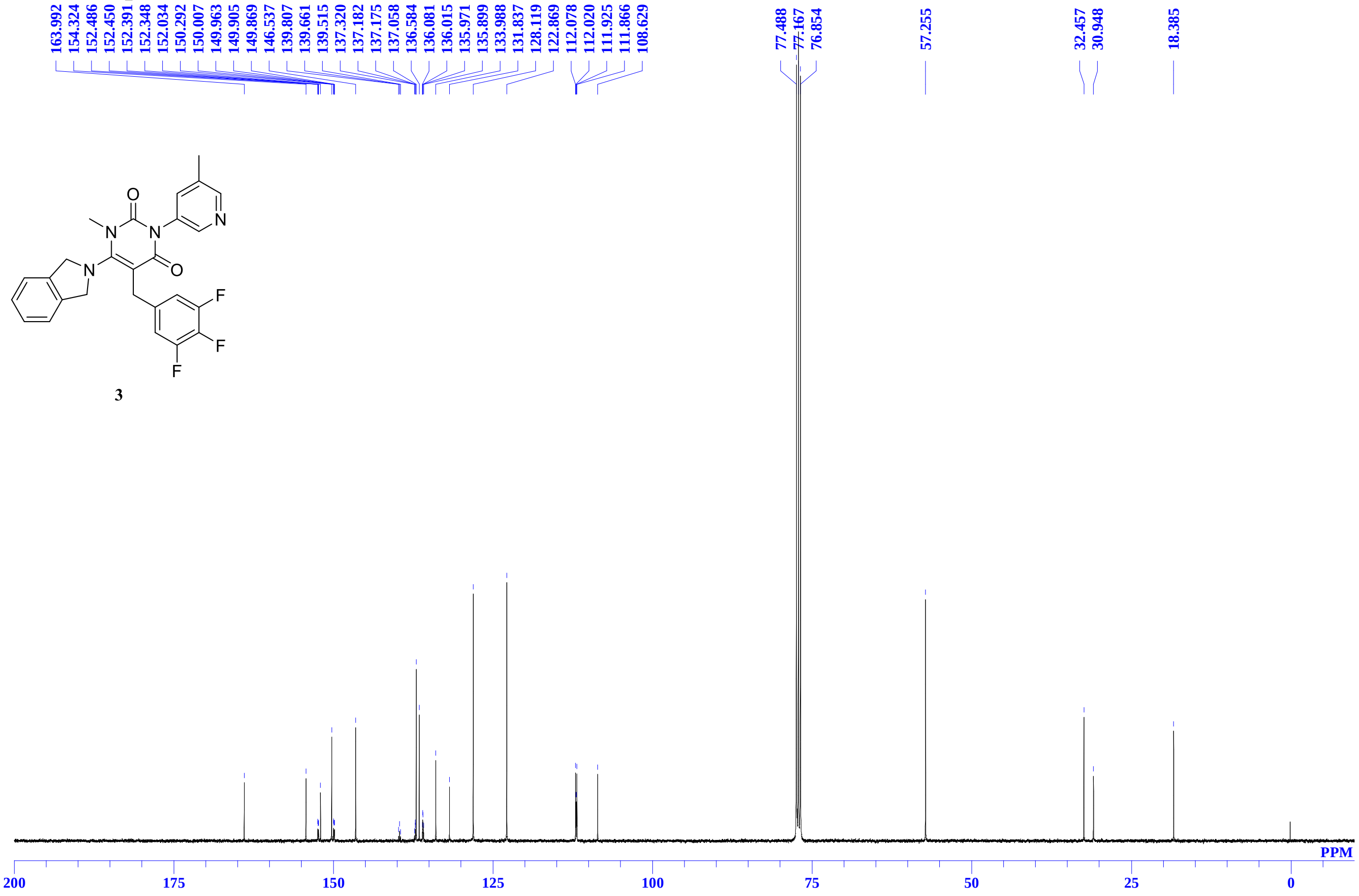

^{1}H NMR spectra of **4** in $CDCl_3$

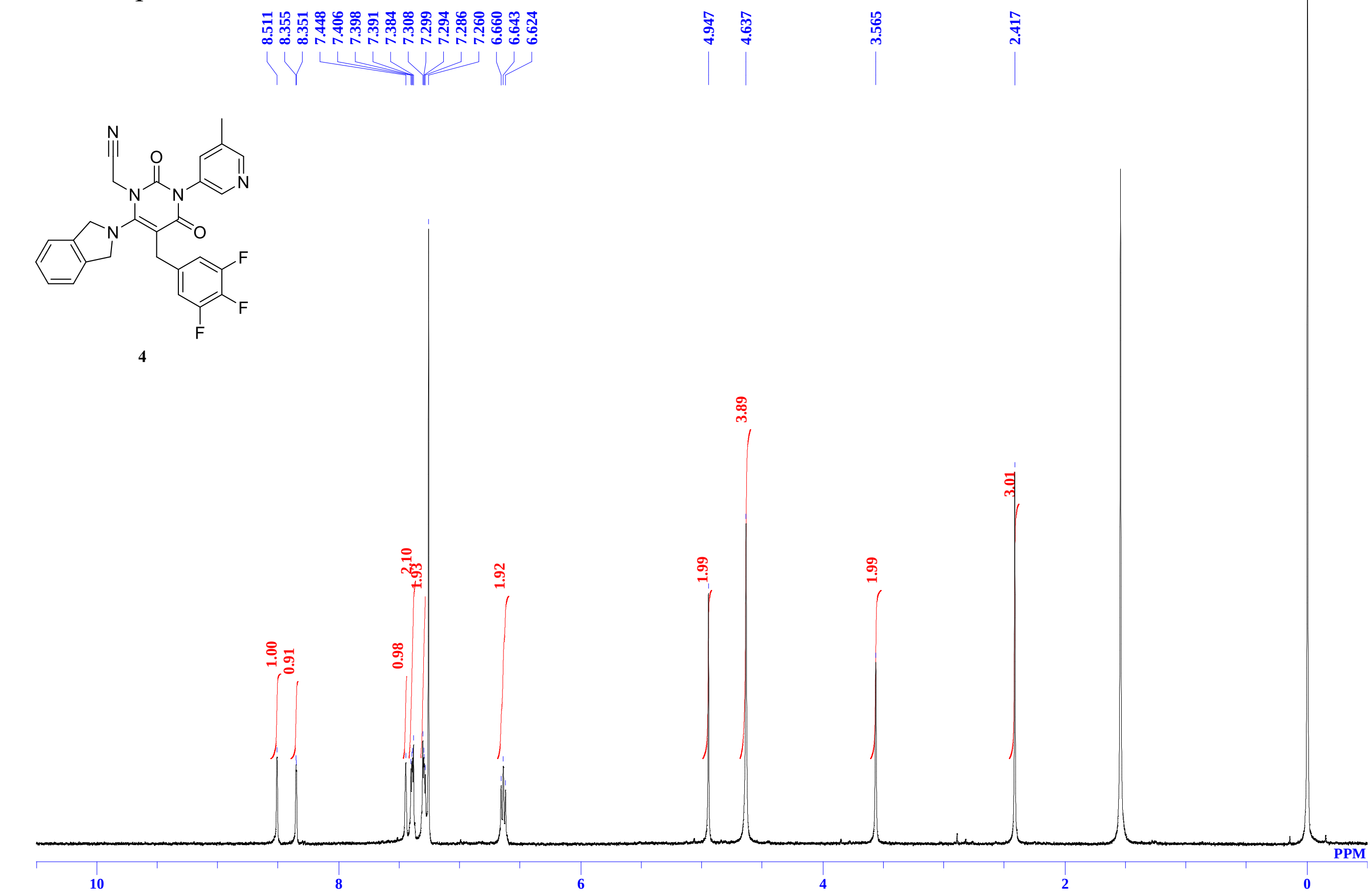

^{13}C NMR spectra of **4** in $CDCl_3$

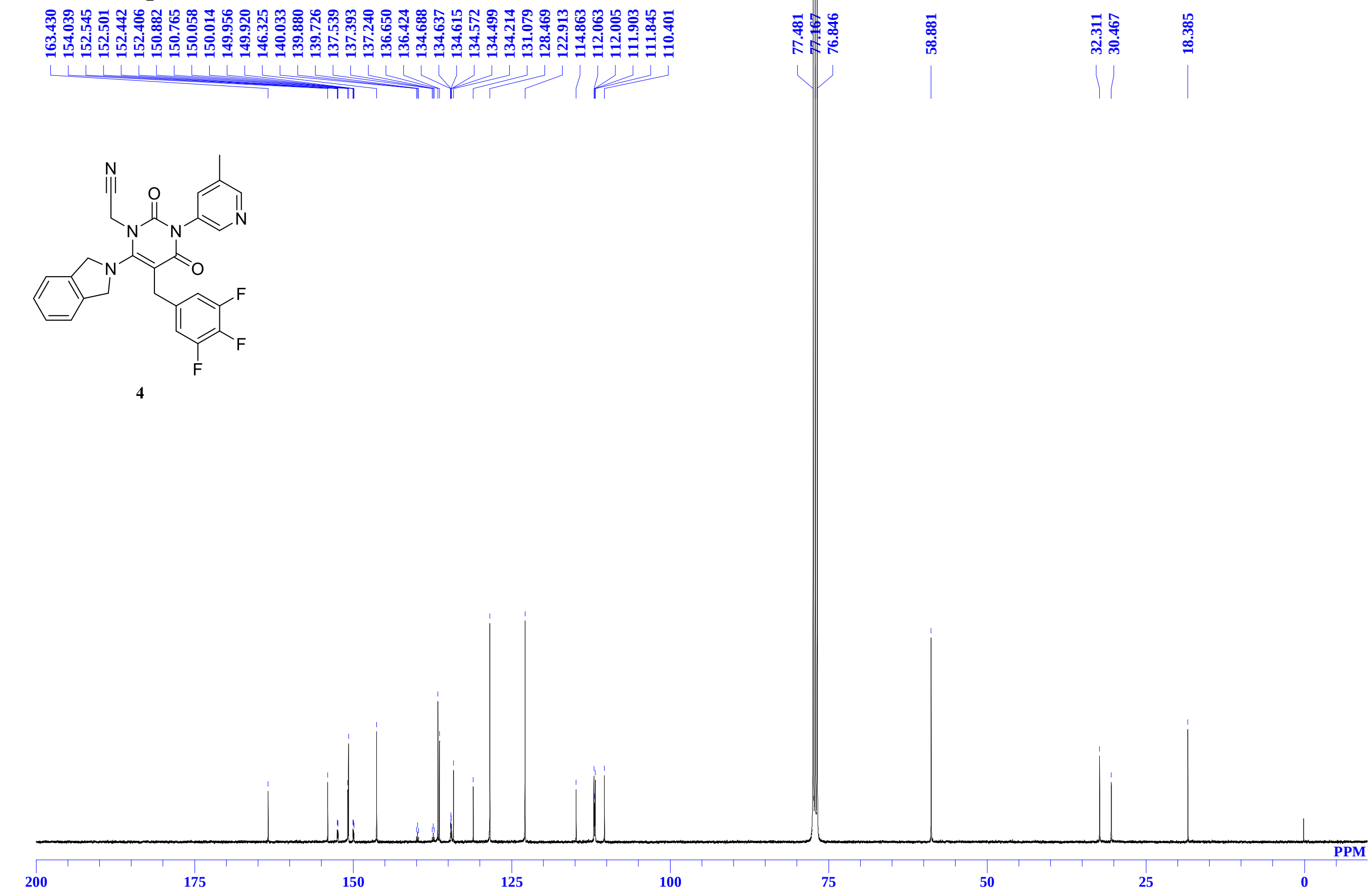

^{1}H NMR spectra of **5** in $CDCl_3$

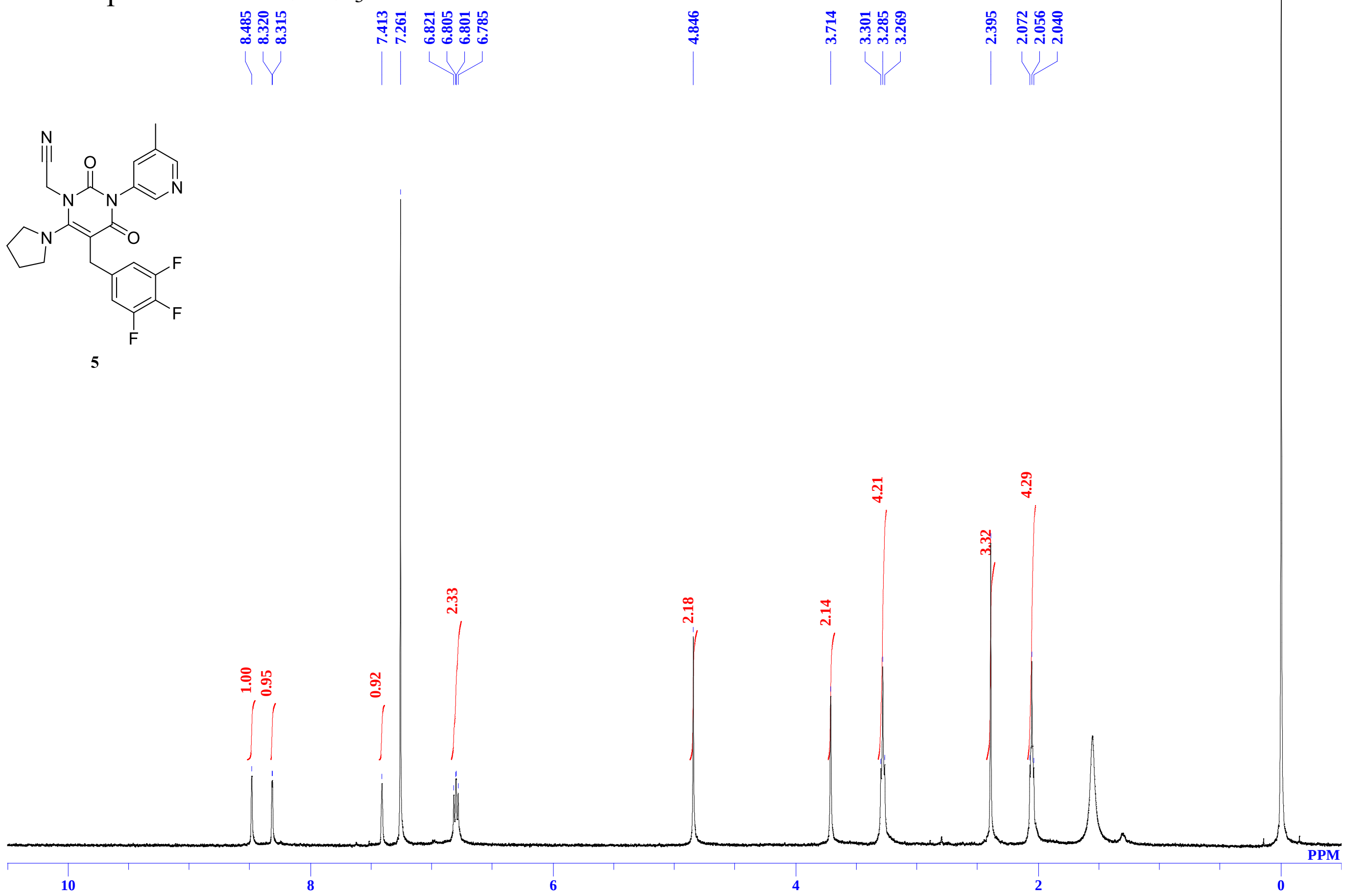

13C NMR spectra of **5** in DMSO-d_6

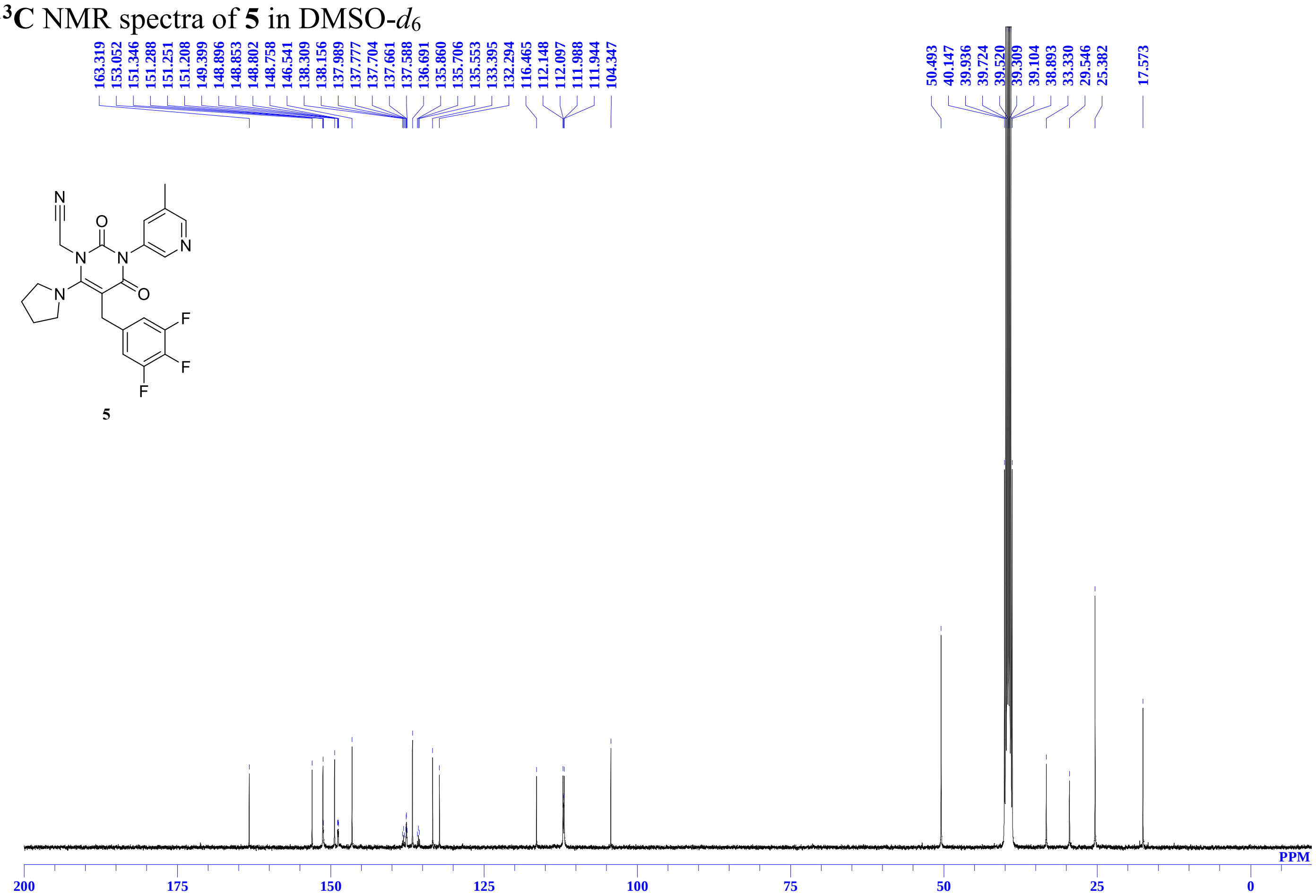

^{1}H NMR spectra of **6** in DMSO-d_6

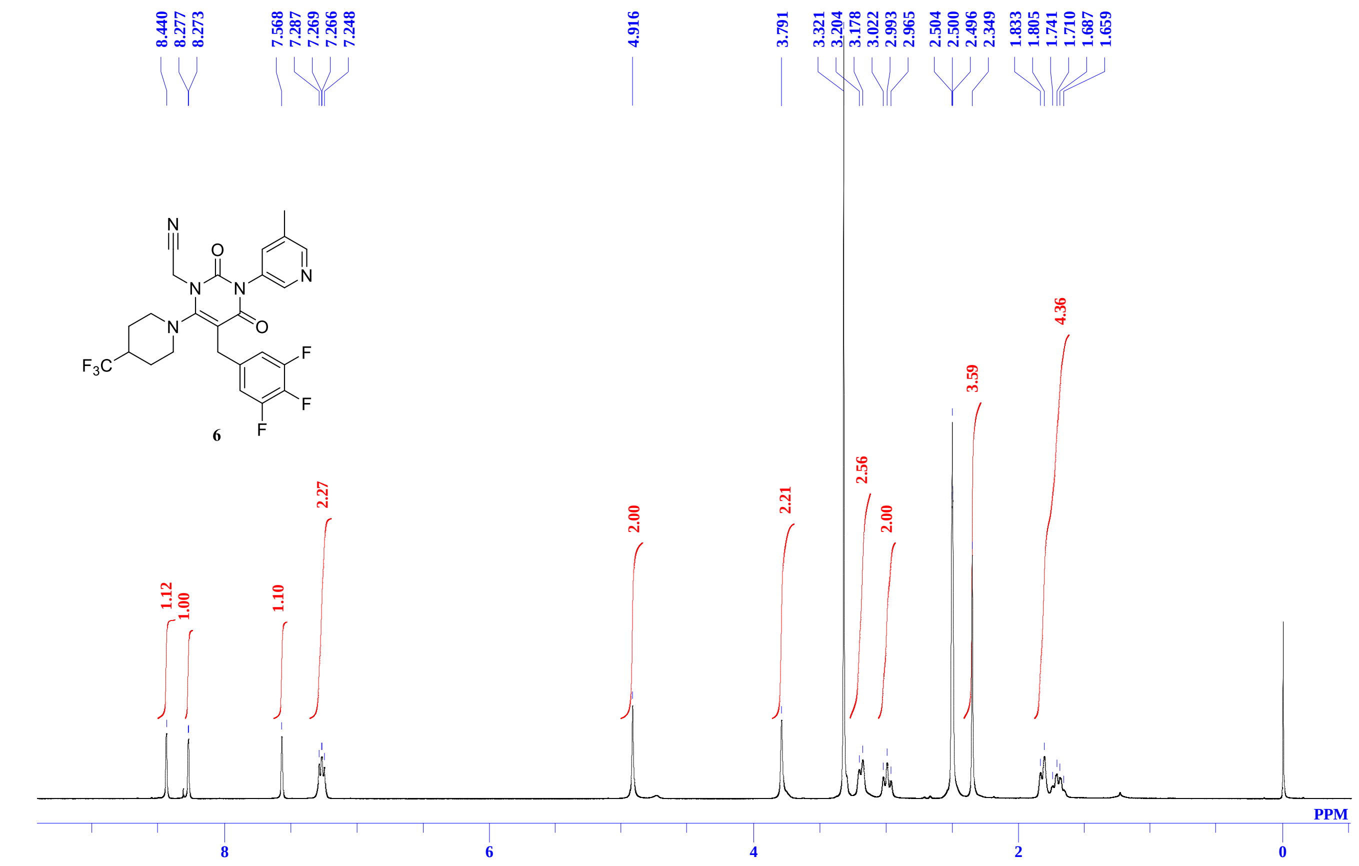

^{13}C NMR spectra of **6** in DMSO-d_6

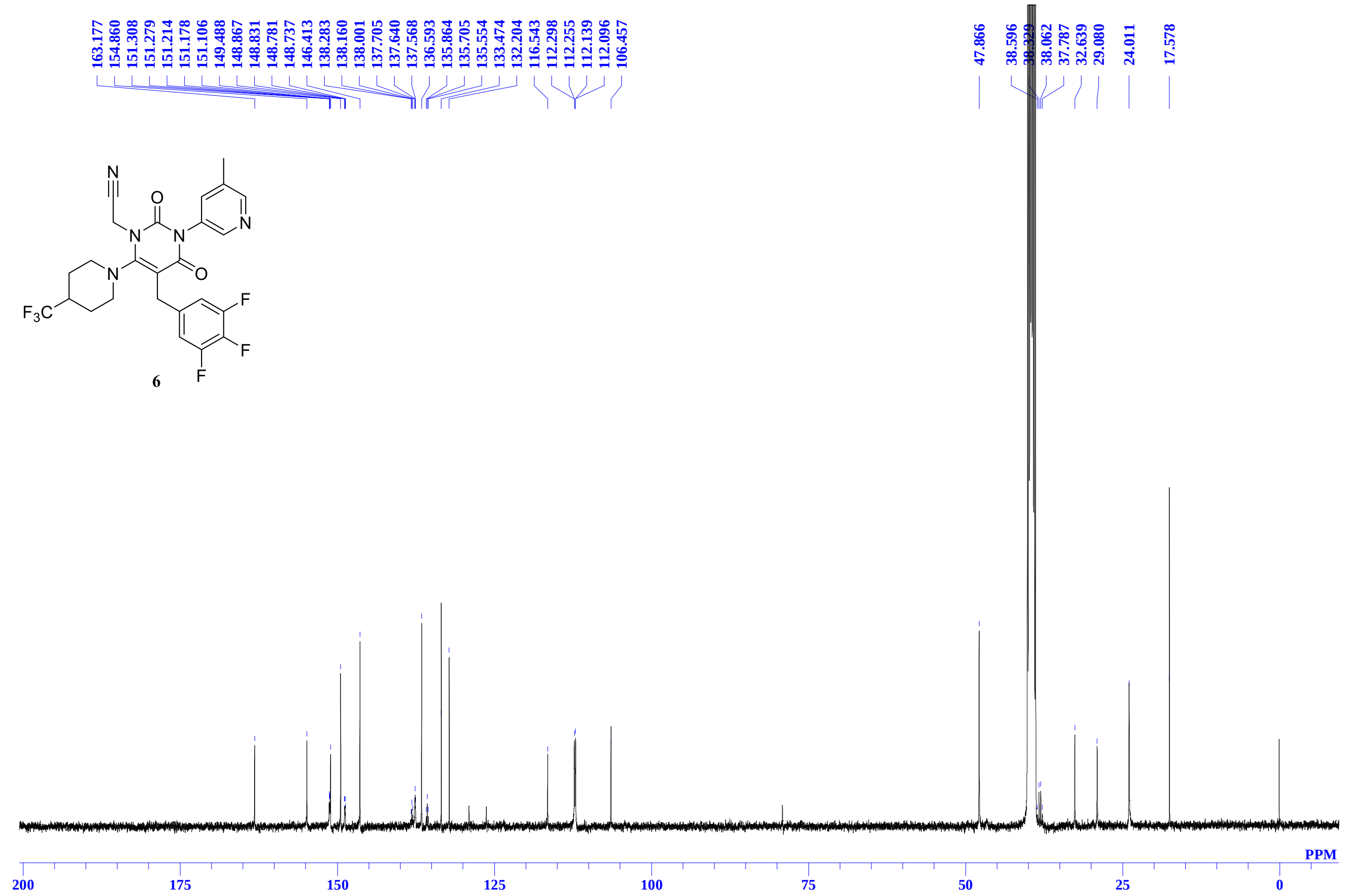

^{1}H NMR spectra of **7** in DMSO-d_6

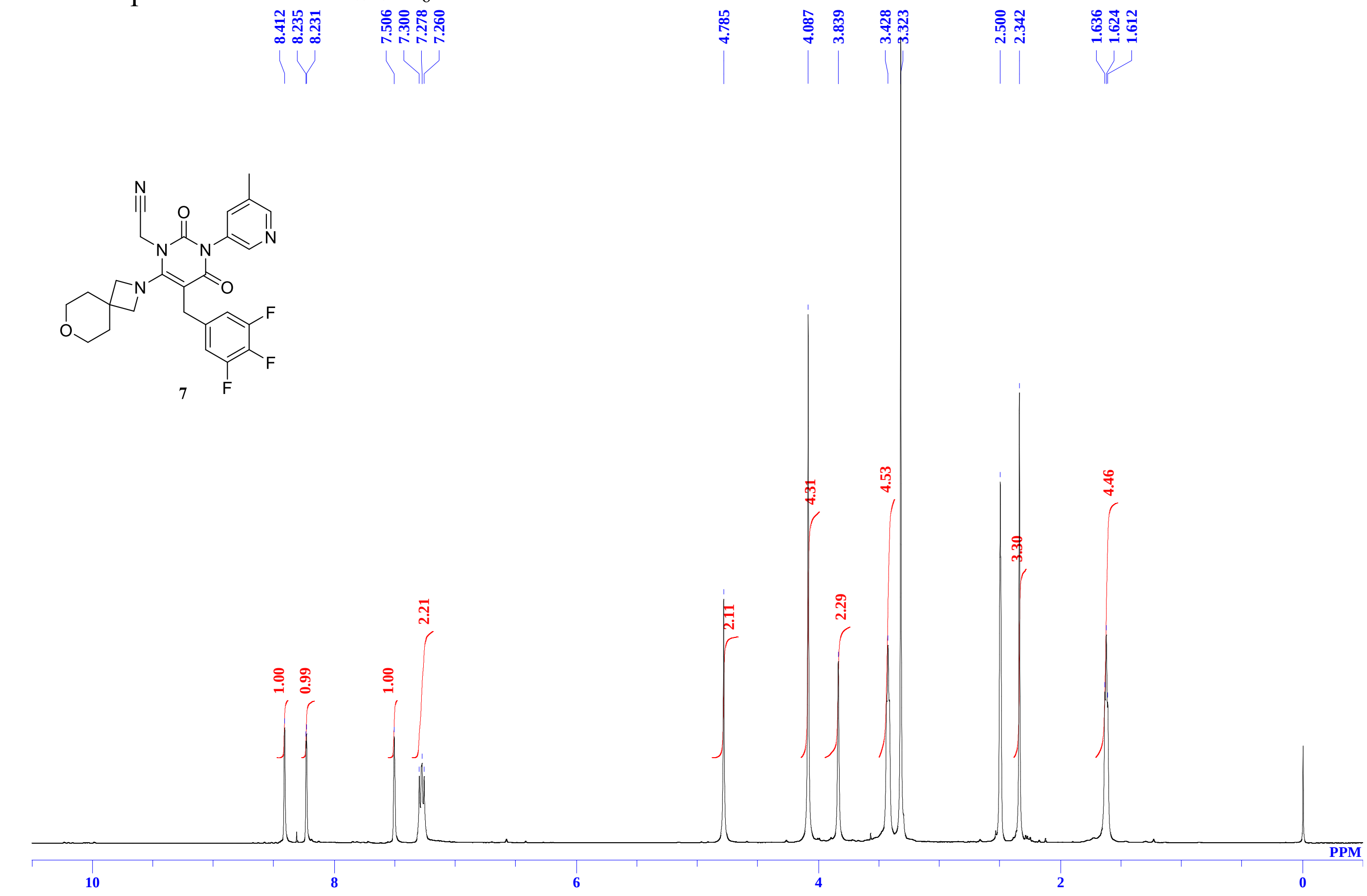

^{13}**C** NMR spectra of **7** in DMSO-d_6

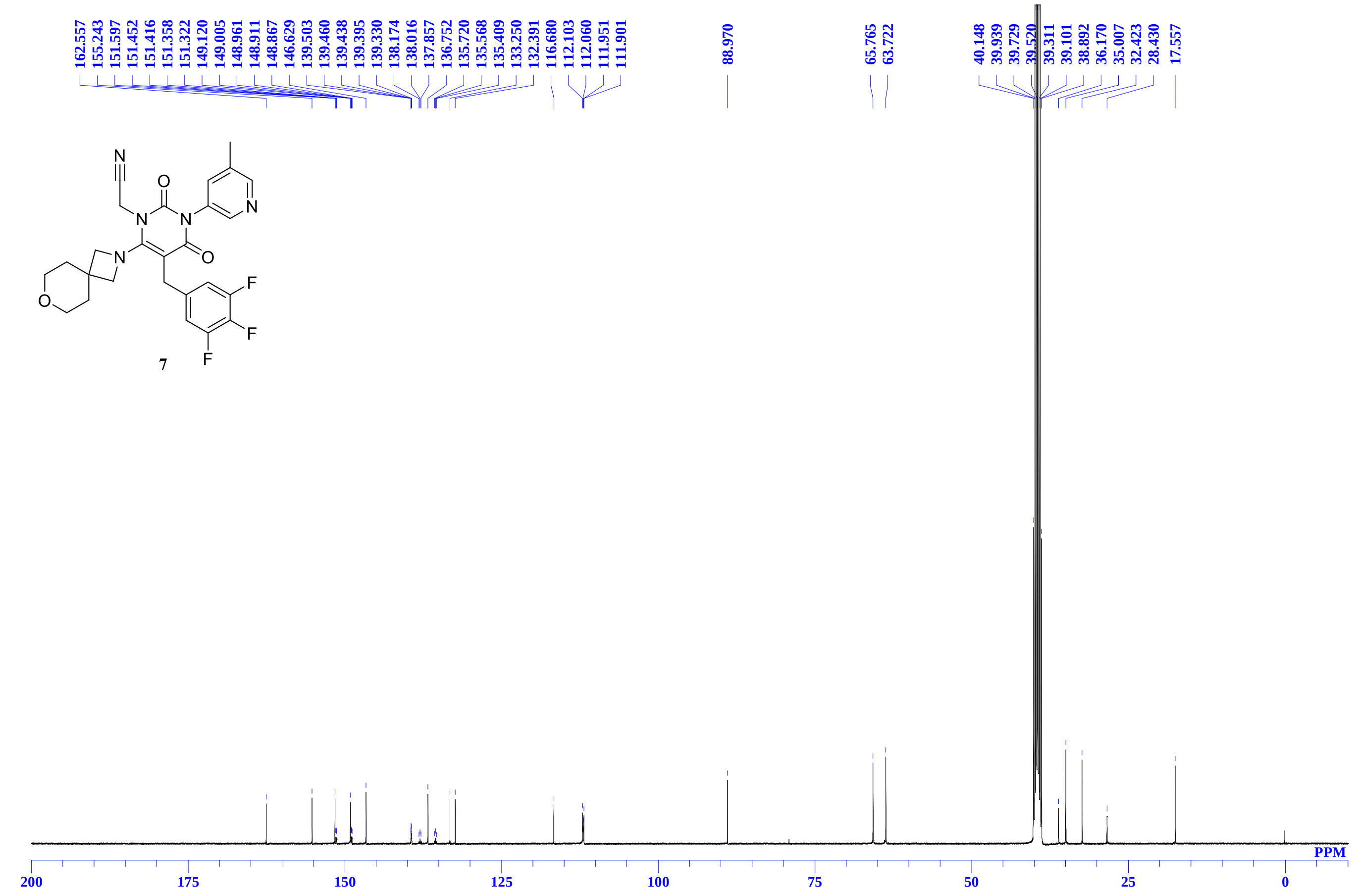

^{1}H NMR spectra of **8** in DMSO-d_6

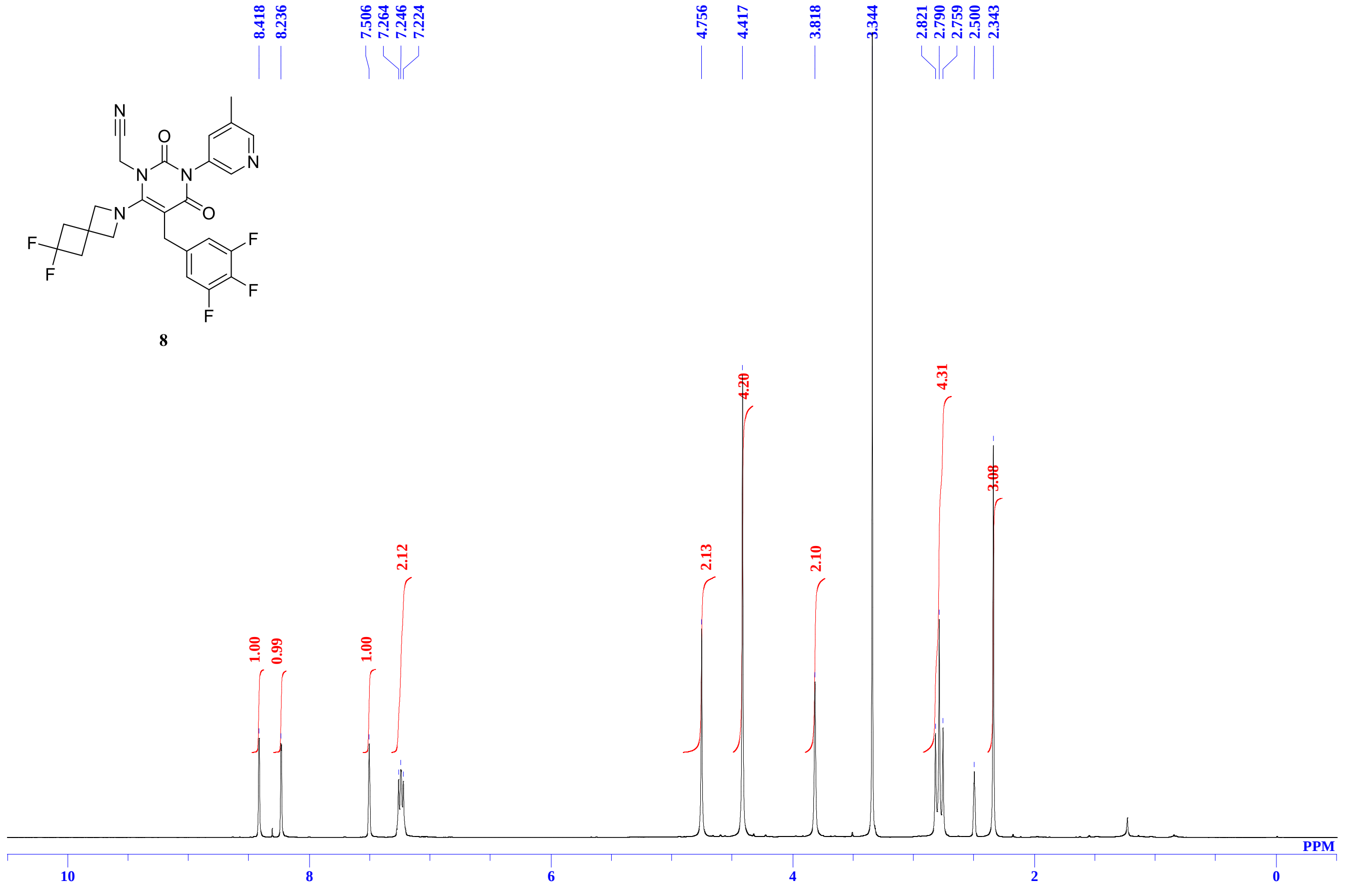

^{13}C NMR spectra of **8** in DMSO-d_6

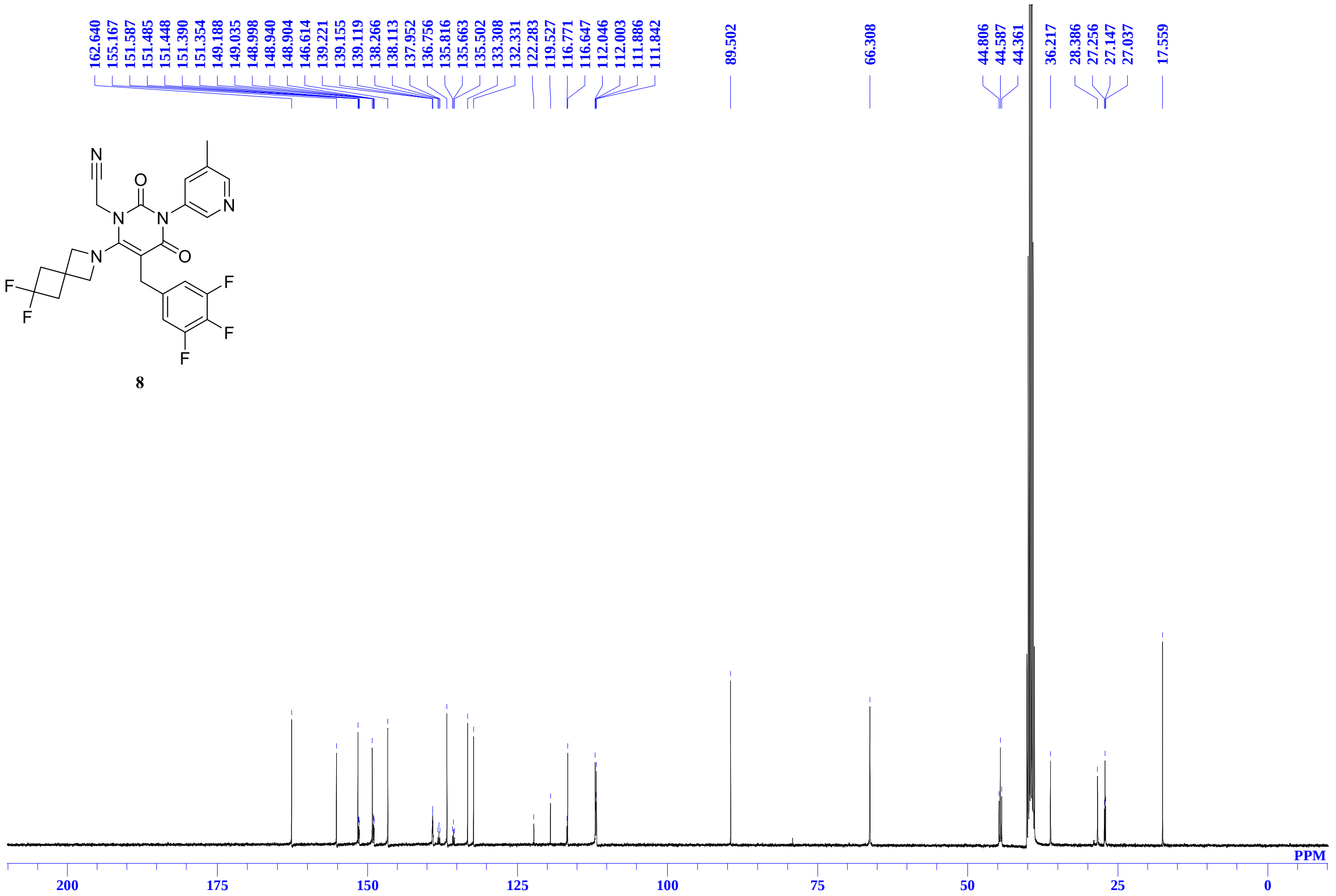

^{1}H NMR spectra of **9** in DMSO-d_6

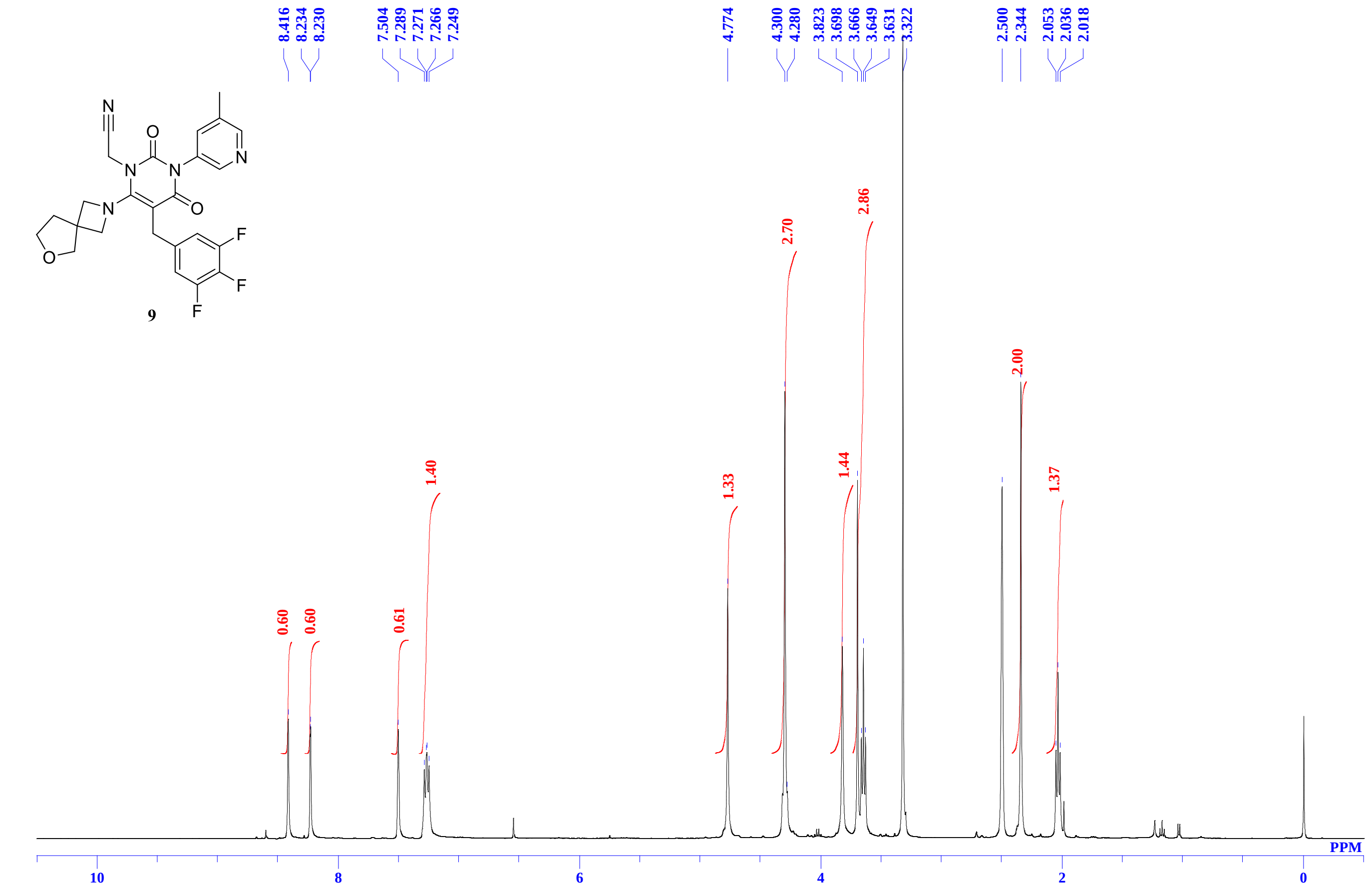

^{13}C NMR spectra of **9** in DMSO-d_6

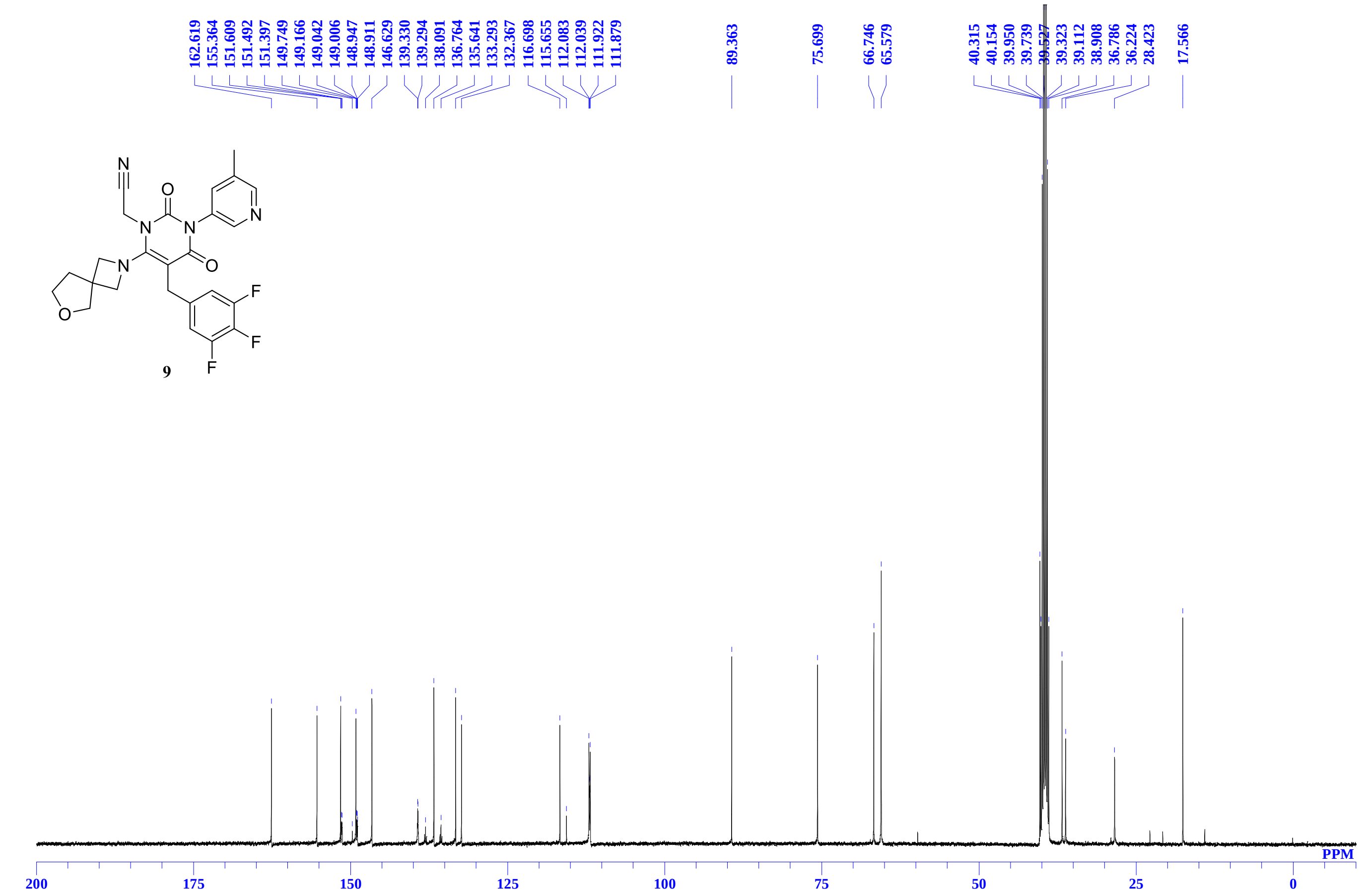

^{1}H NMR spectra of **10** in DMSO-d_6

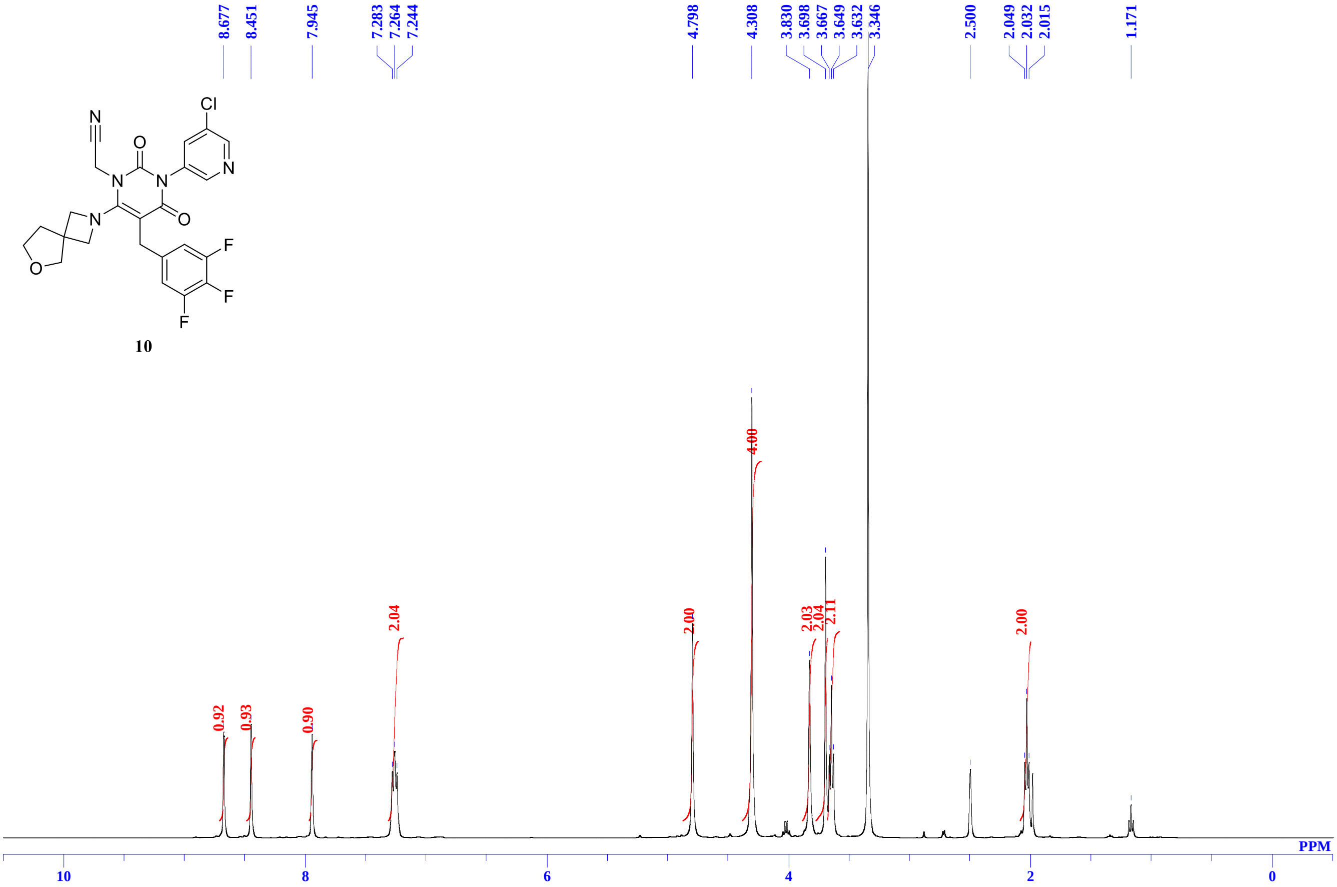

^{13}C NMR spectra of **10** in DMSO-d_6

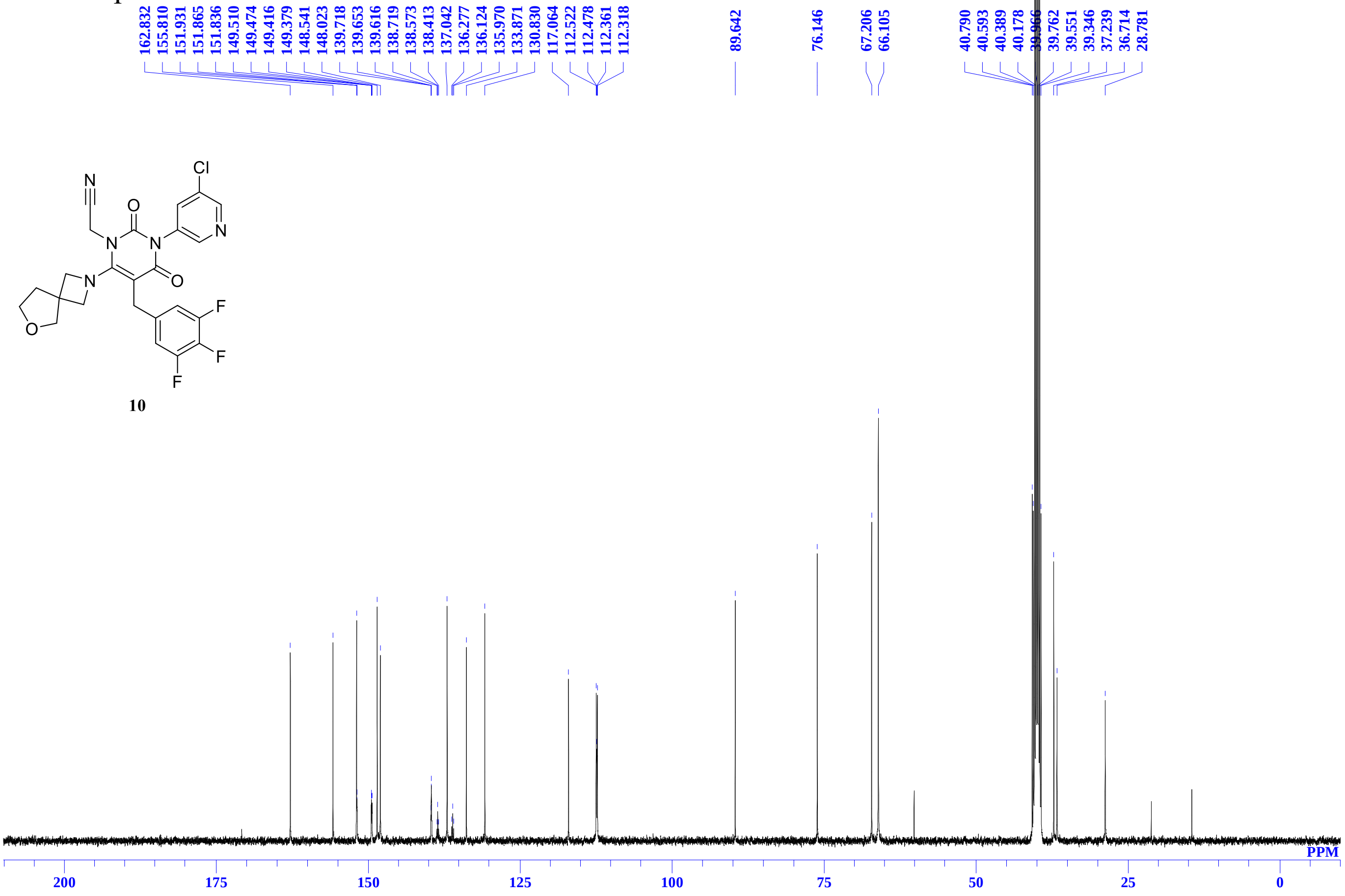

^{1}H NMR spectra of **11** in $CDCl_3$

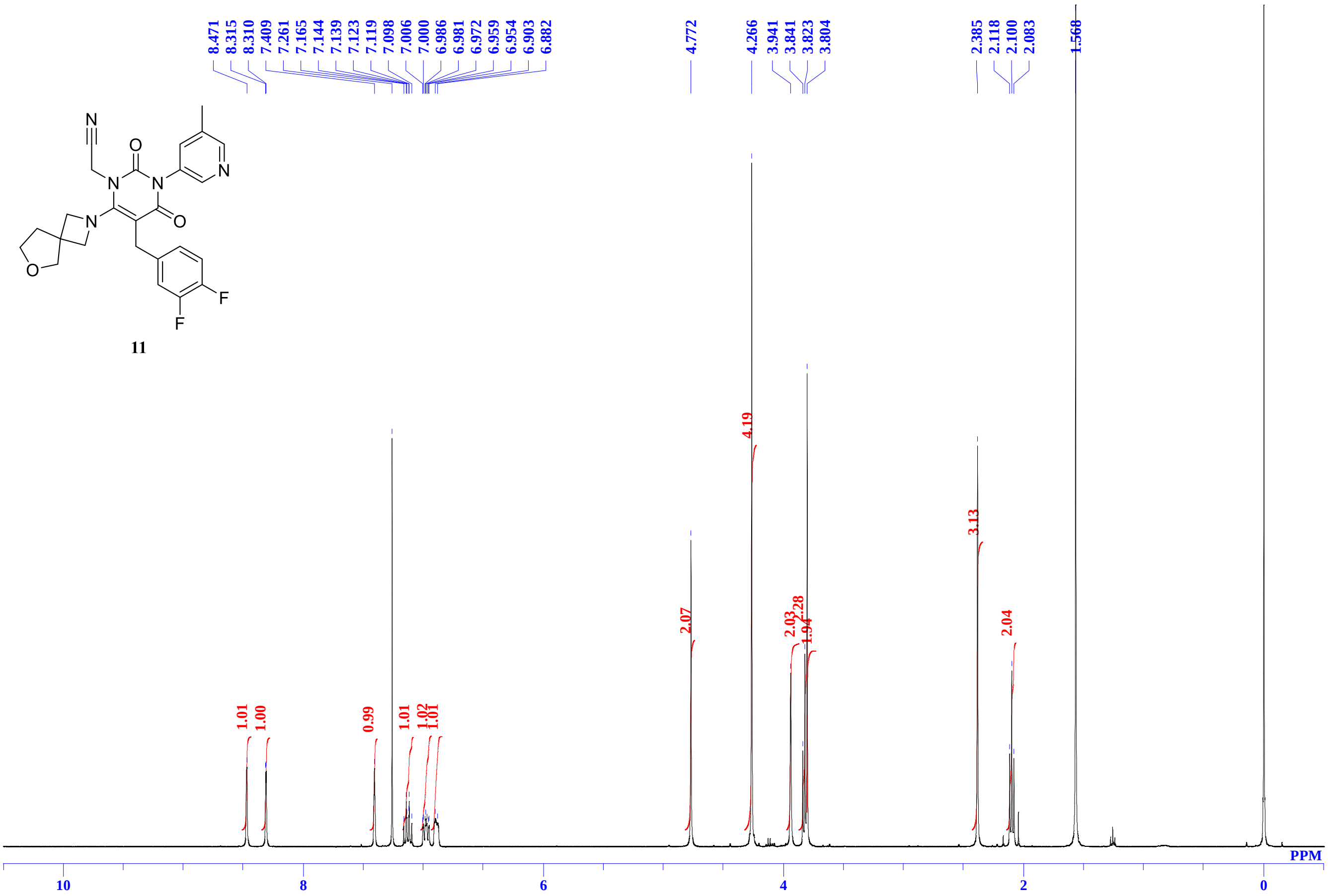

^{13}C NMR spectra of **11** in DMSO-d_6

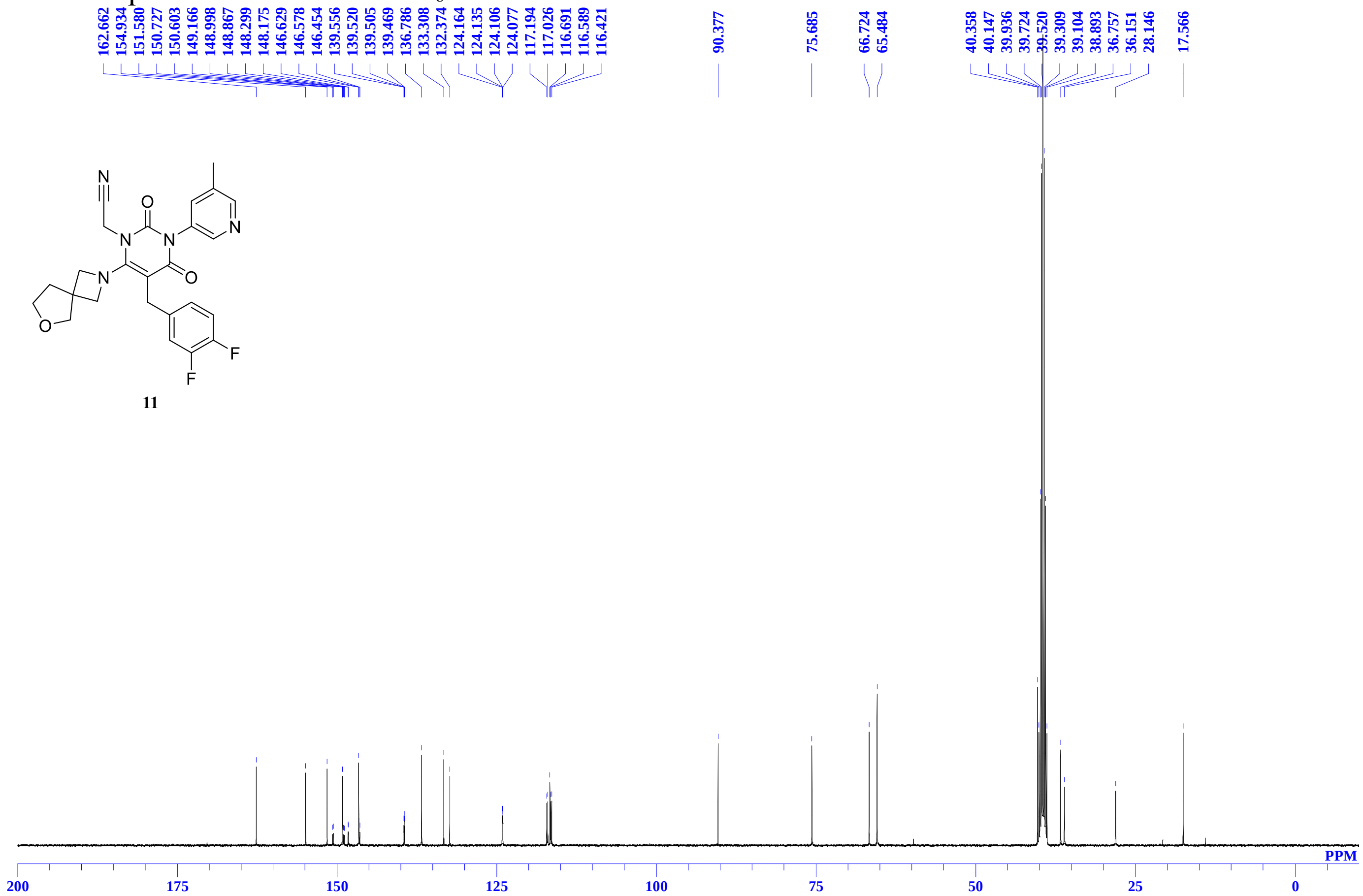

^{1}H NMR spectra of **12** in $CDCl_3$

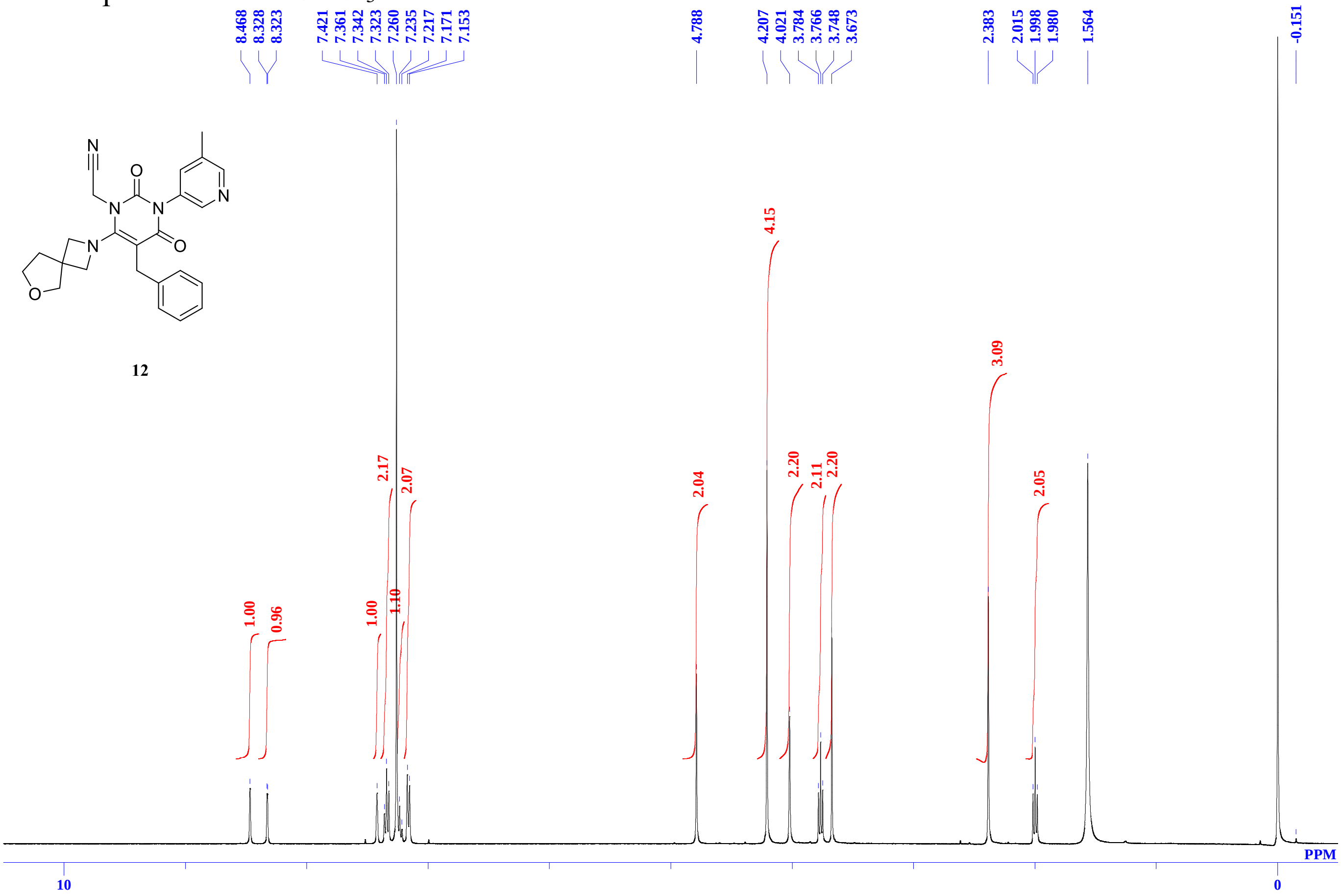

^{13}C NMR spectra of **12** in DMSO-d_6

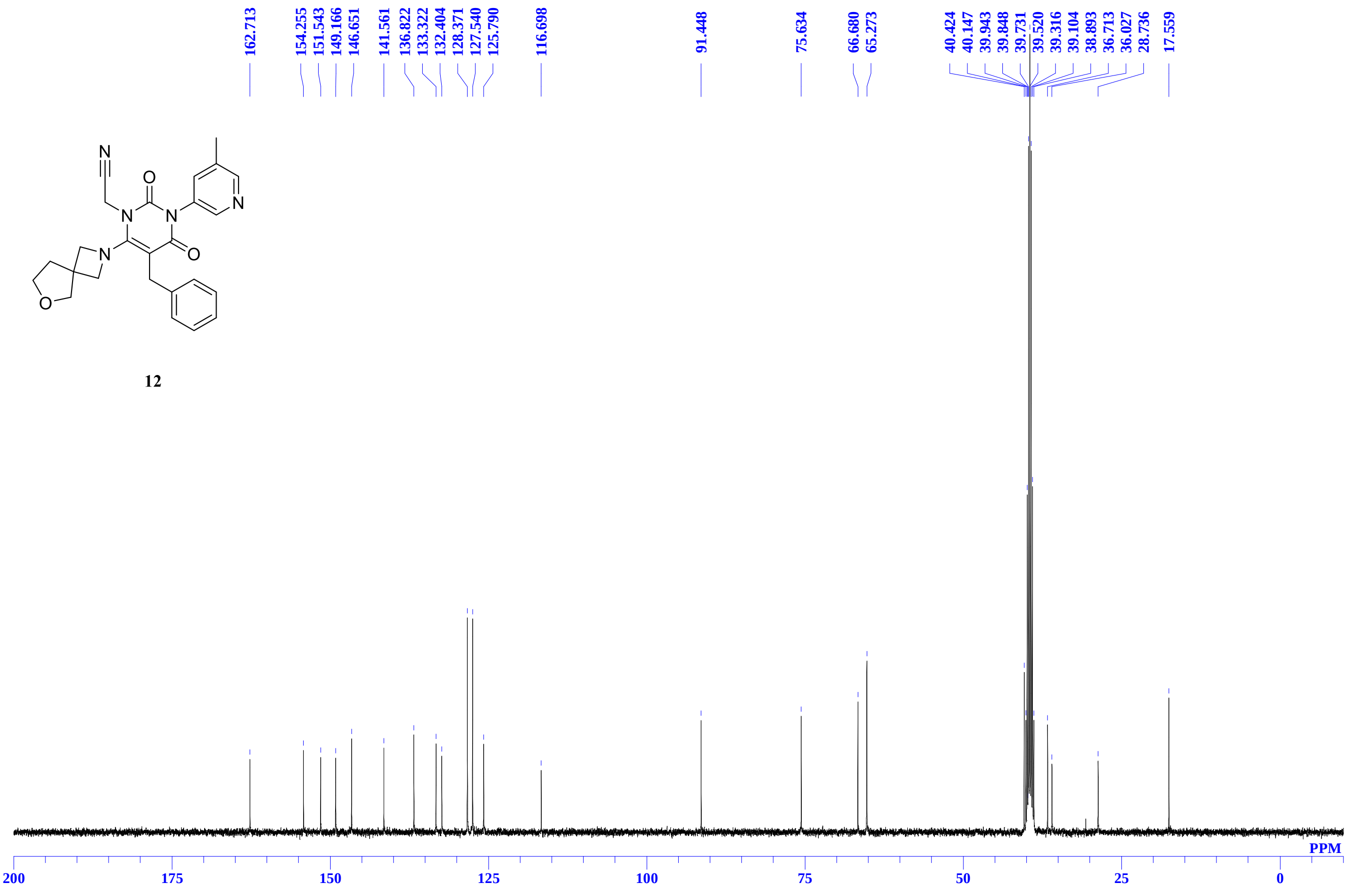

^{1}H NMR spectra of **13** in $CDCl_3$

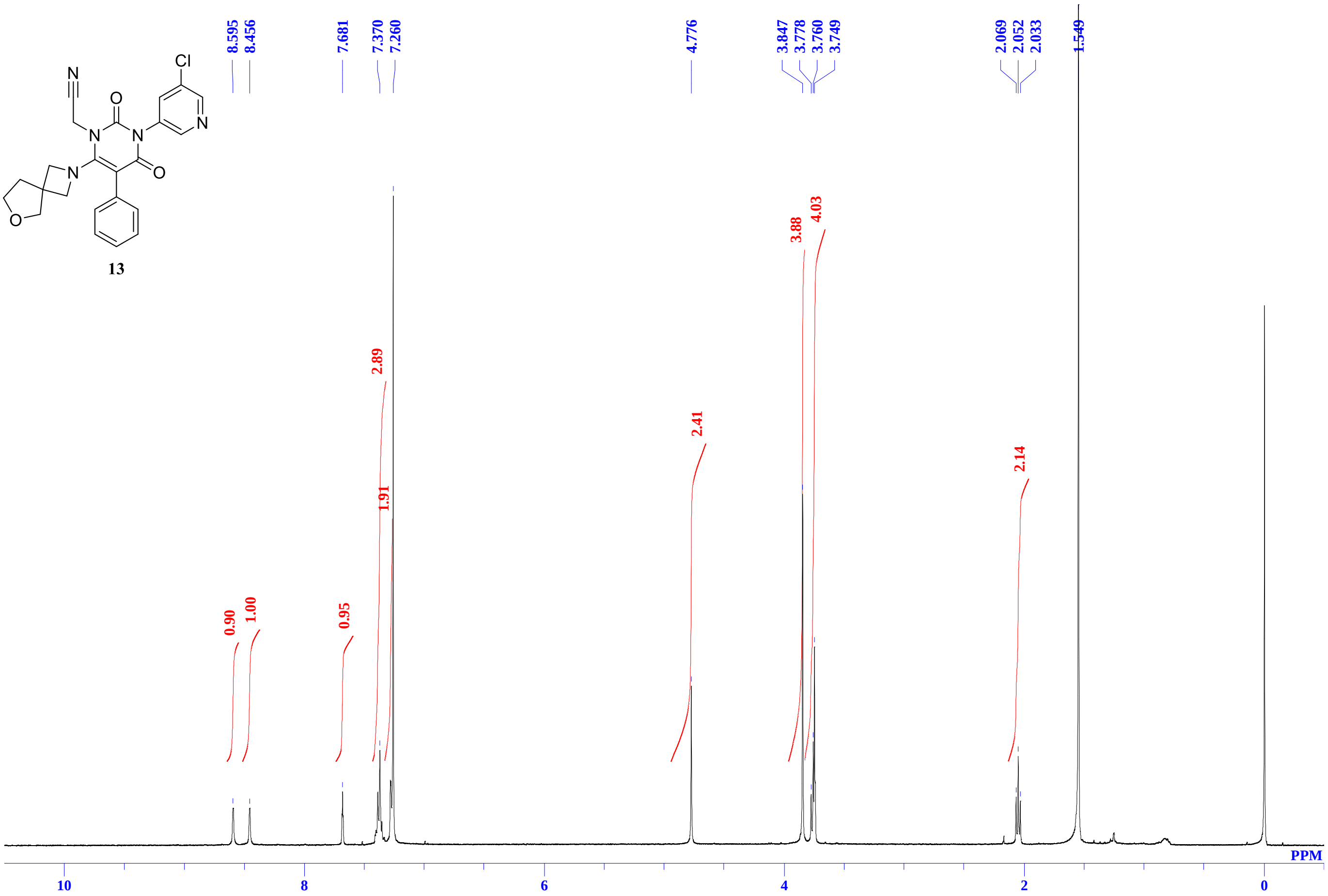

^{13}C NMR spectra of **13** in $CDCl_3$

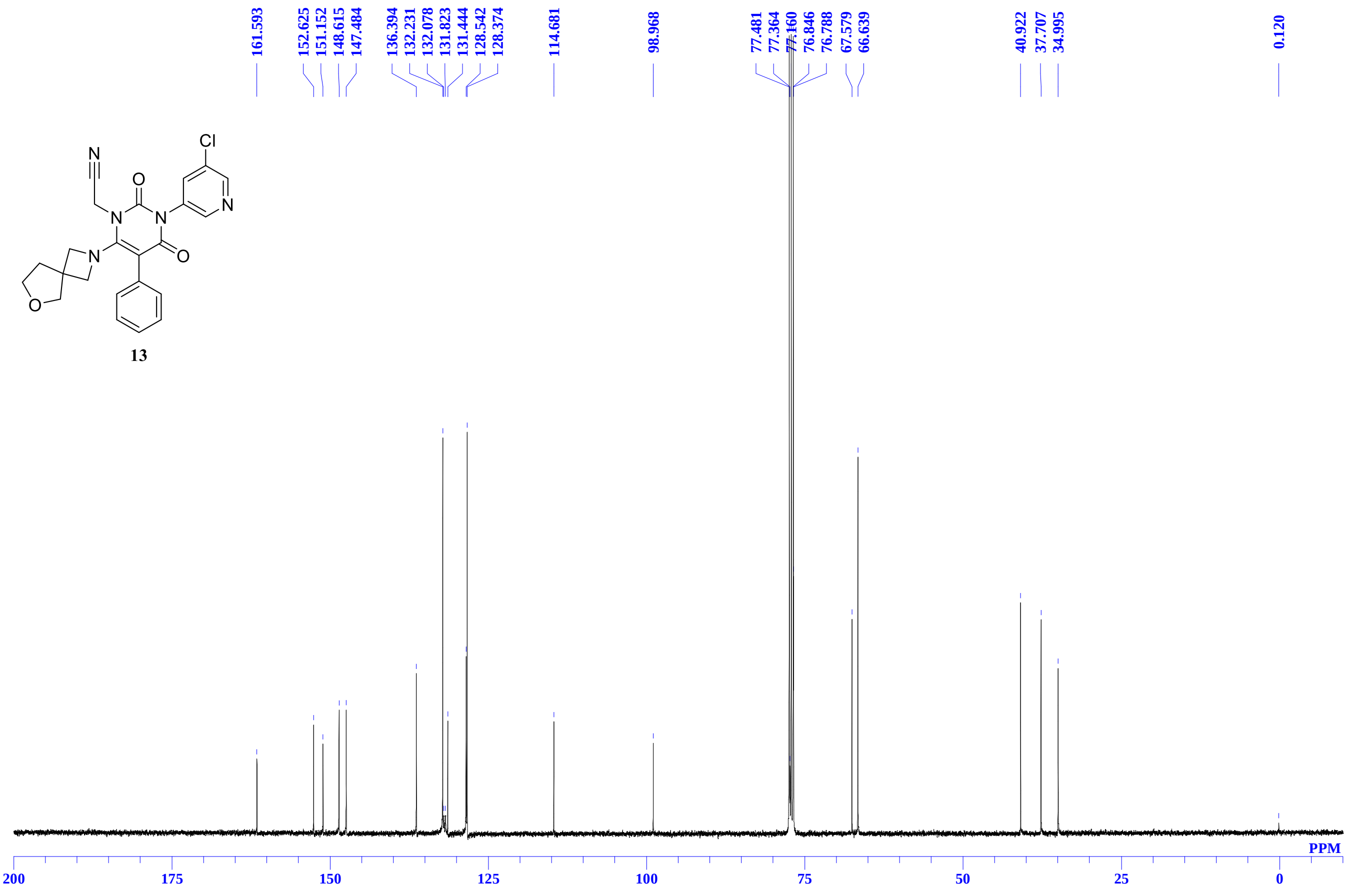

^{1}H NMR spectra of **14** in $CDCl_3$

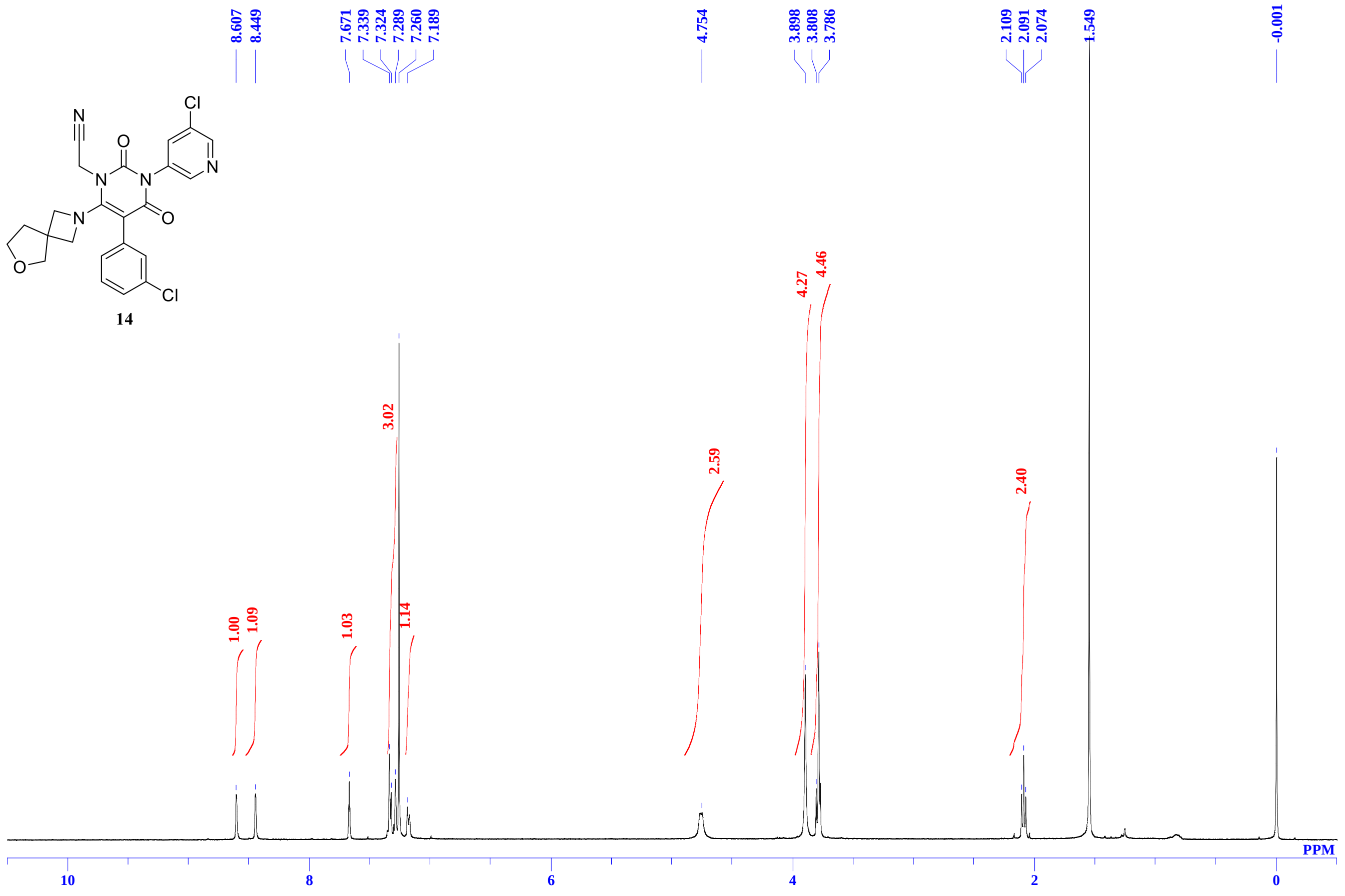

^{13}C NMR spectra of **14** in $CDCl_3$

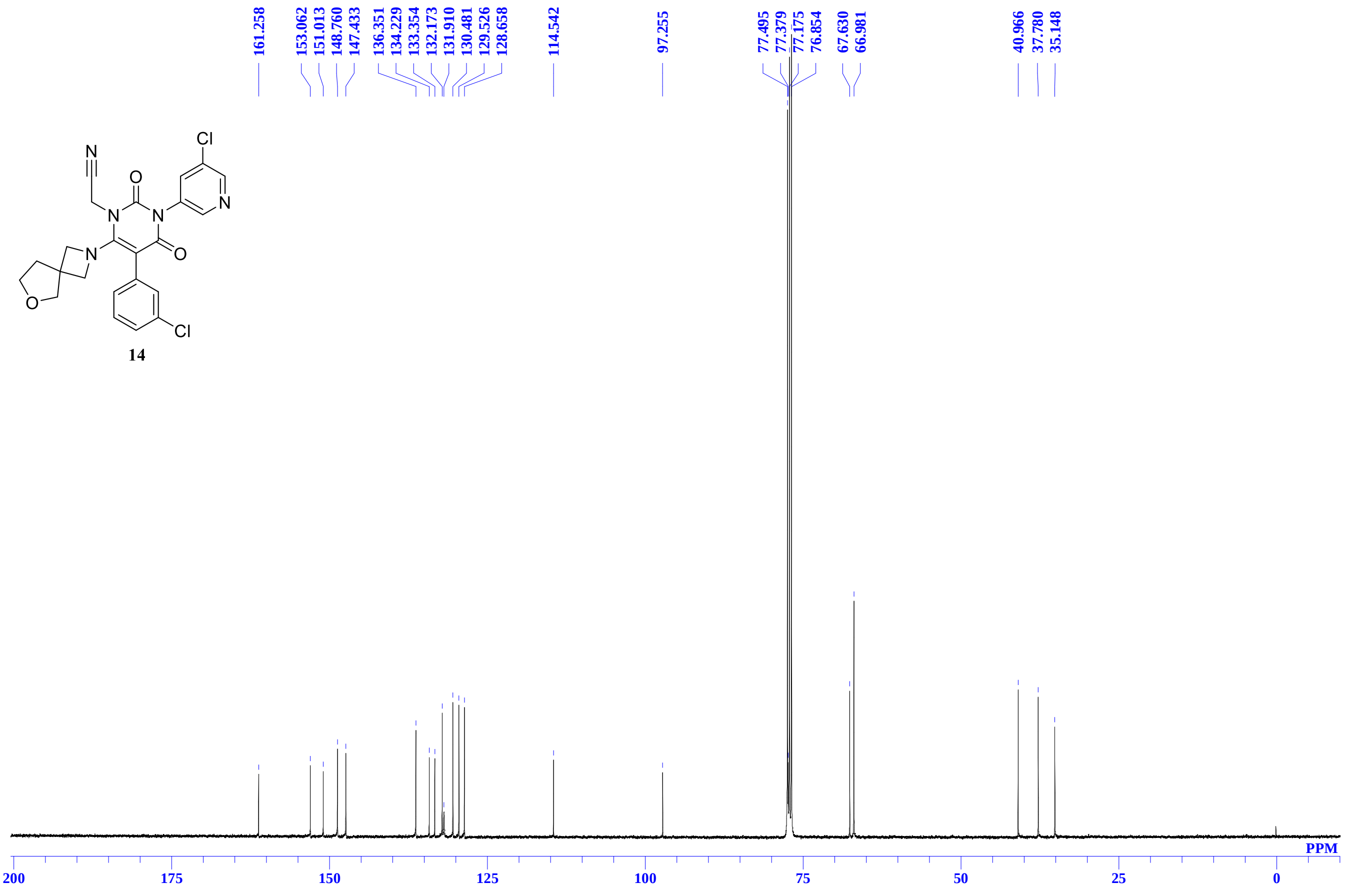

^{1}H NMR spectra of **15** in DMSO-d_6

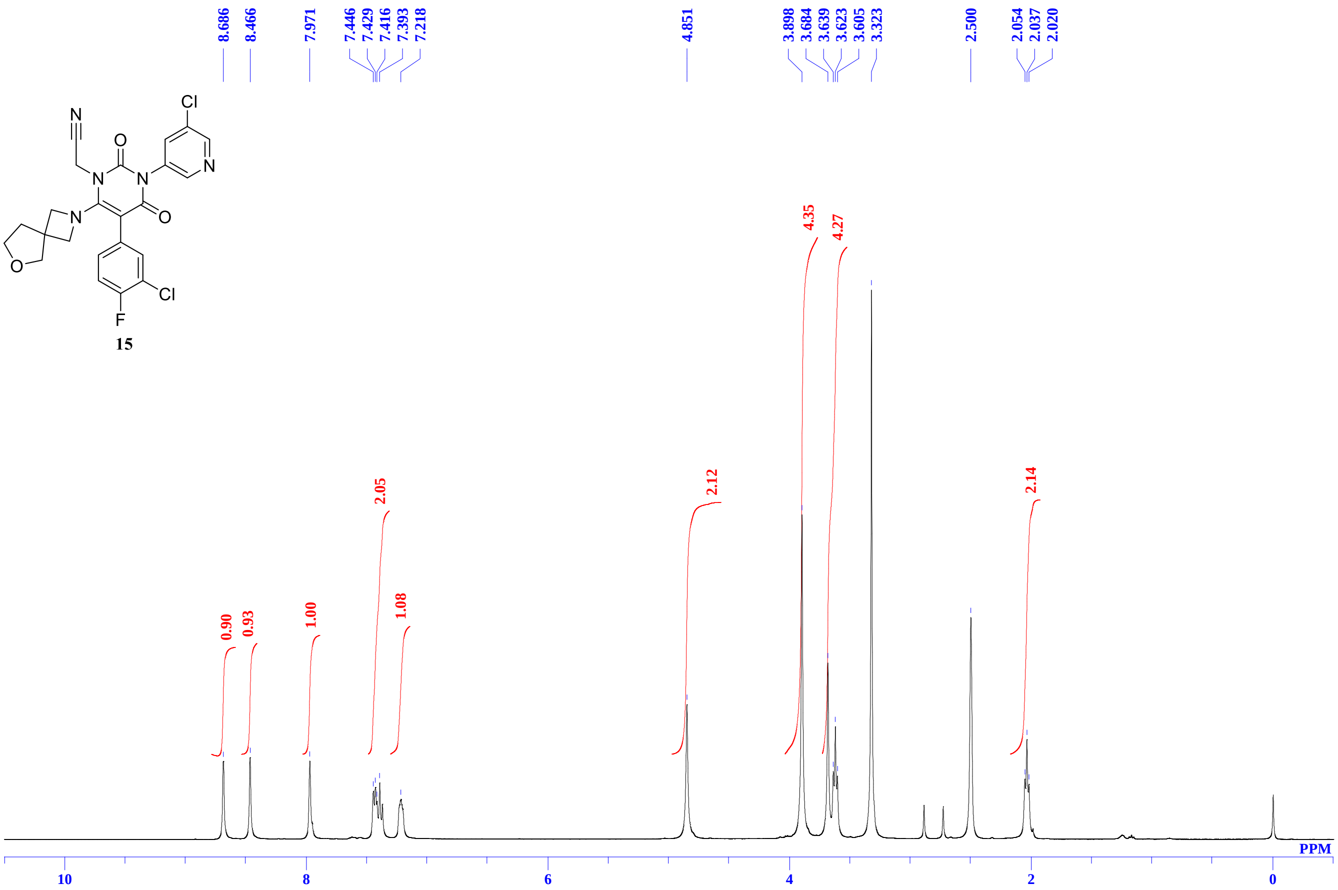

^{13}C NMR spectra of **15** in DMSO-d_6

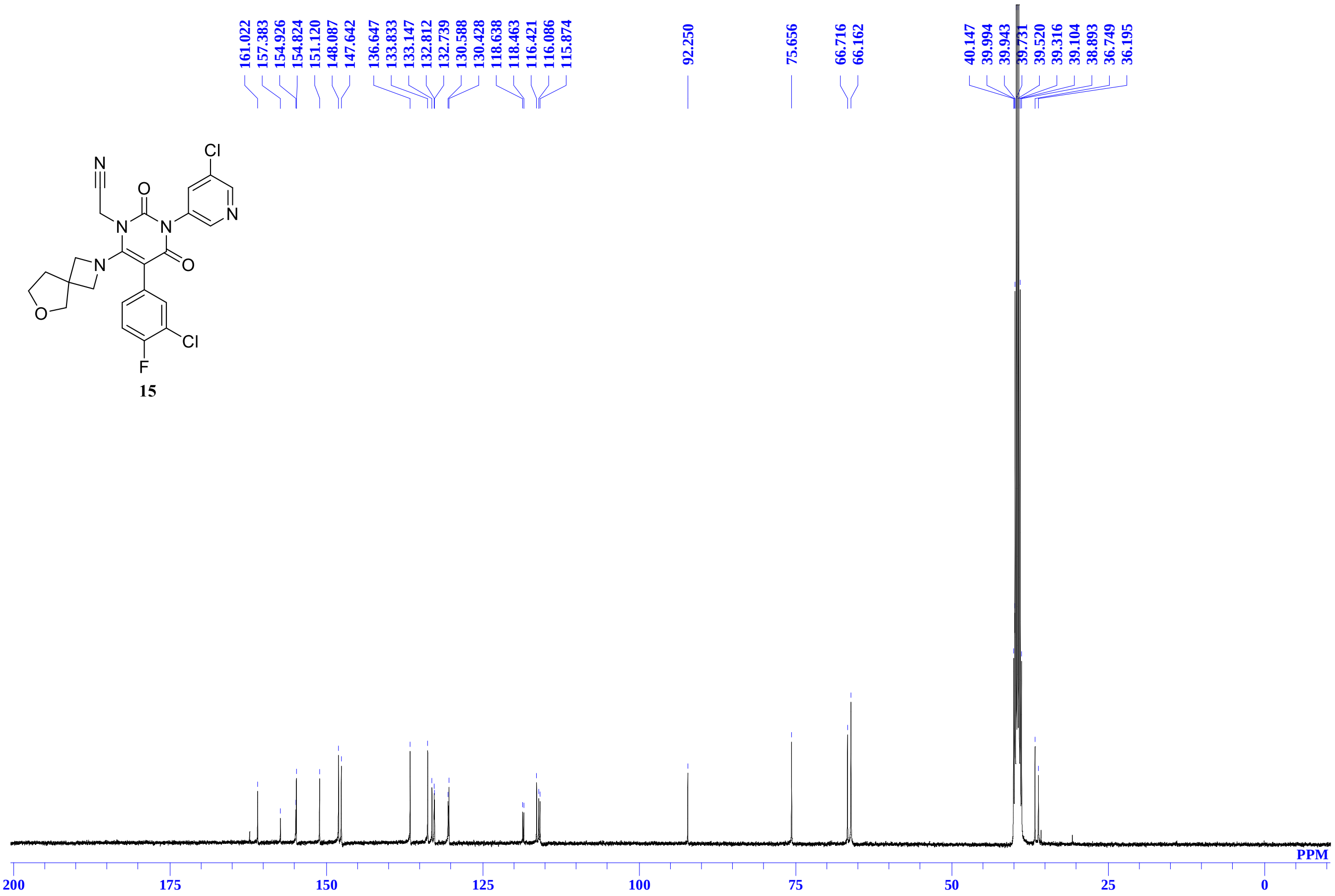

^{1}H NMR spectra of **16** in DMSO-d_6

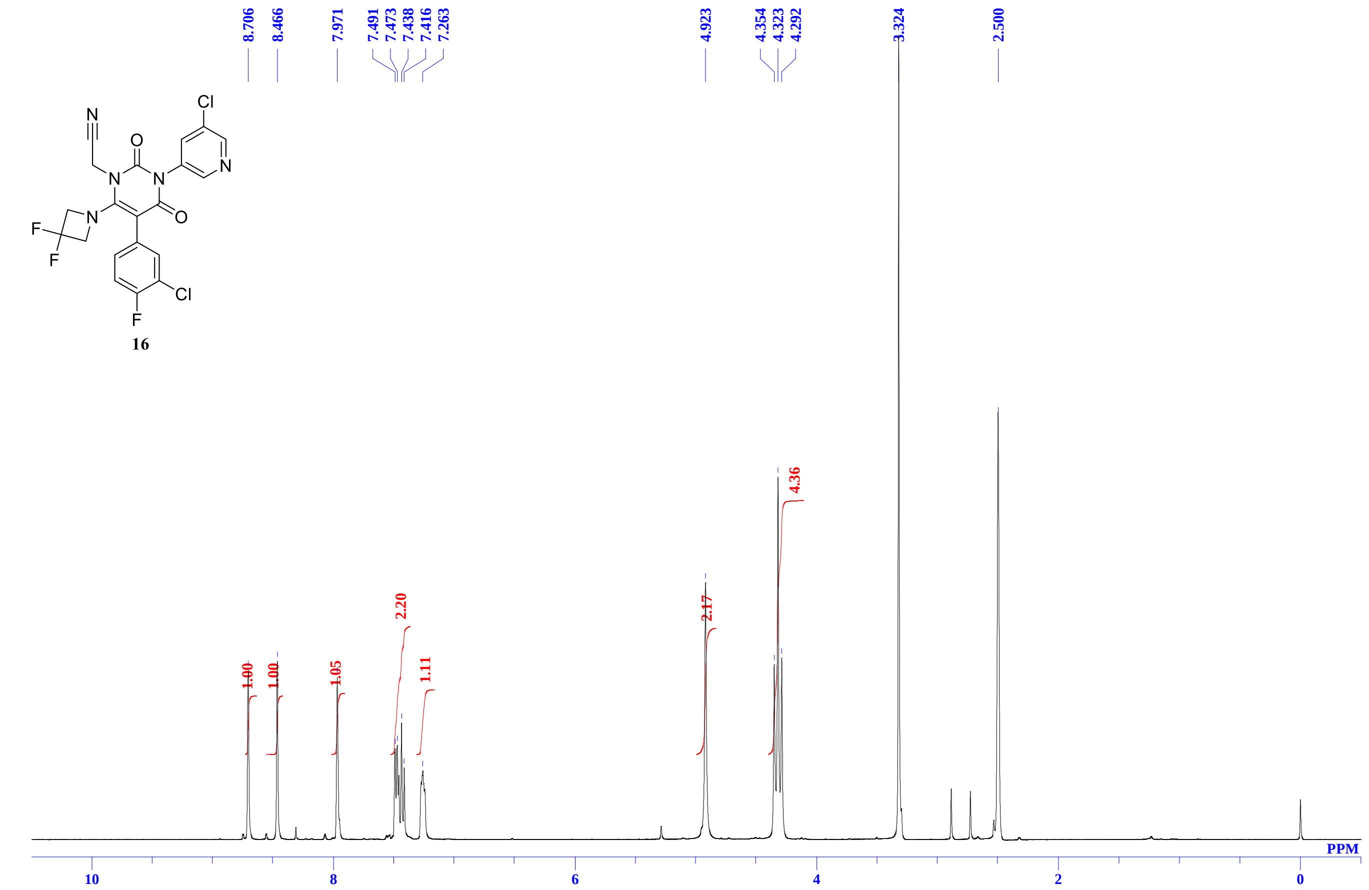

^{13}**C** NMR spectra of **16** in DMSO-d_6

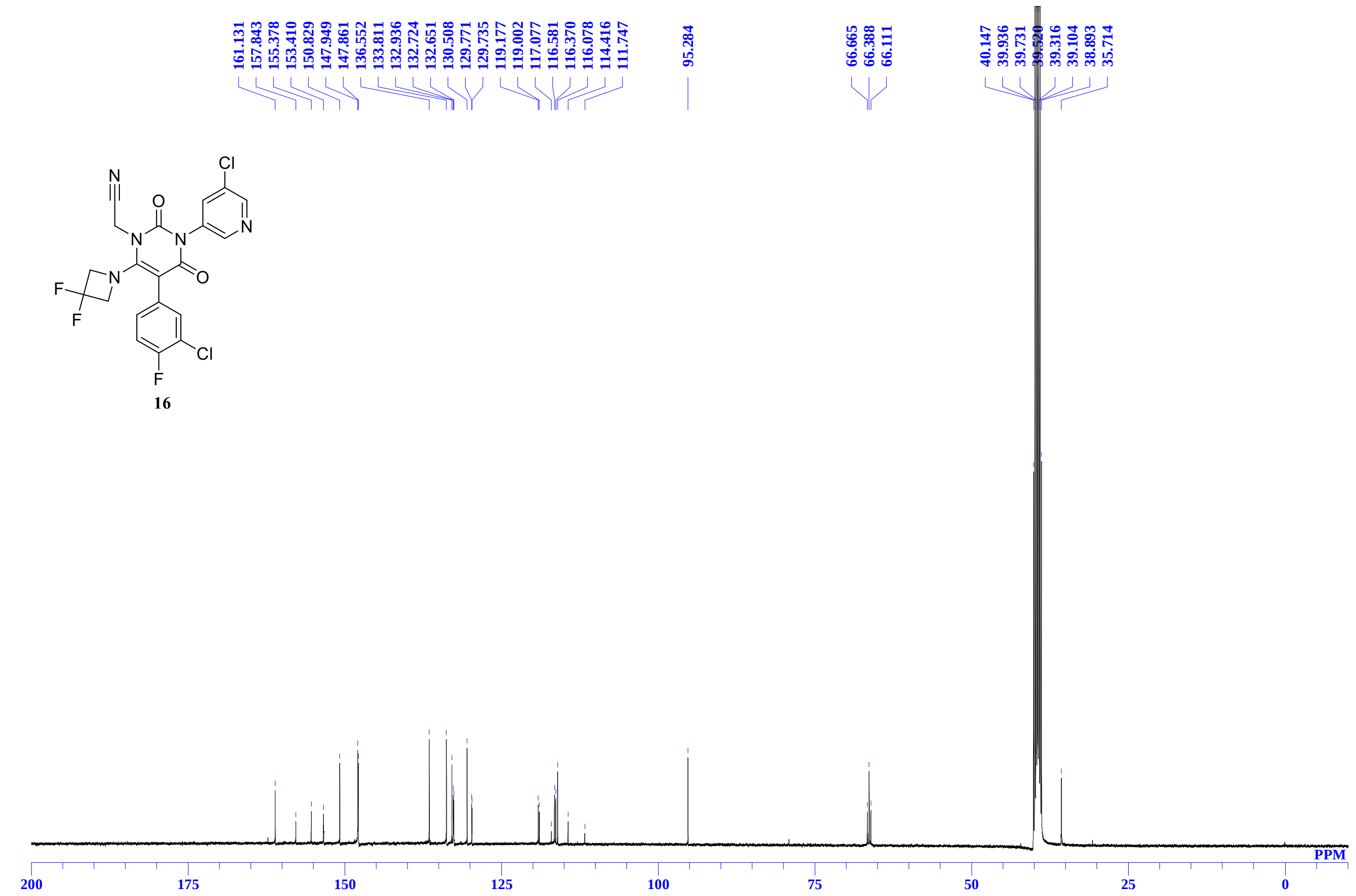

^{1}H NMR spectra of **17** in DMSO-d_6

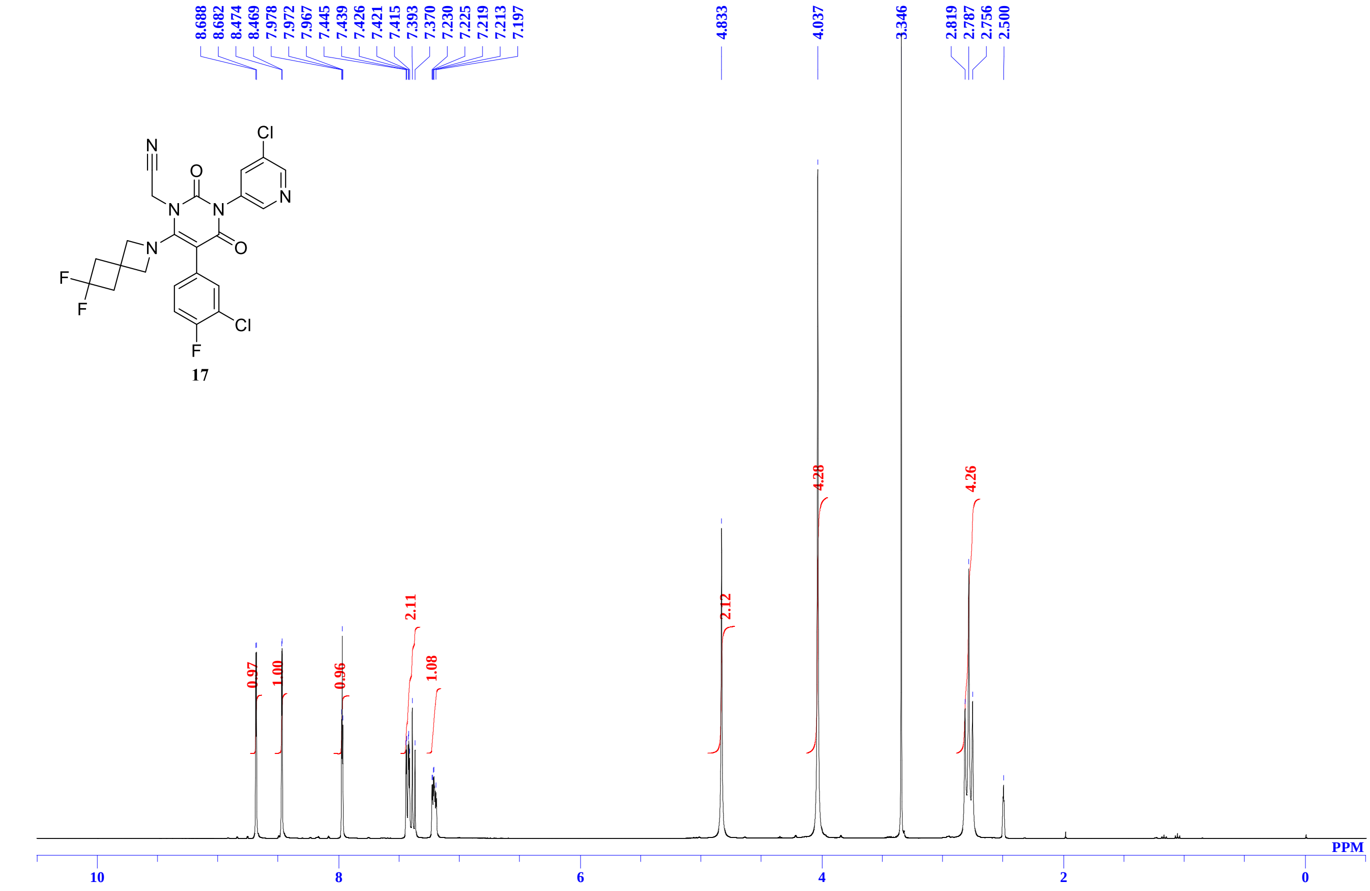

^{13}C NMR spectra of **17** in DMSO-d_6

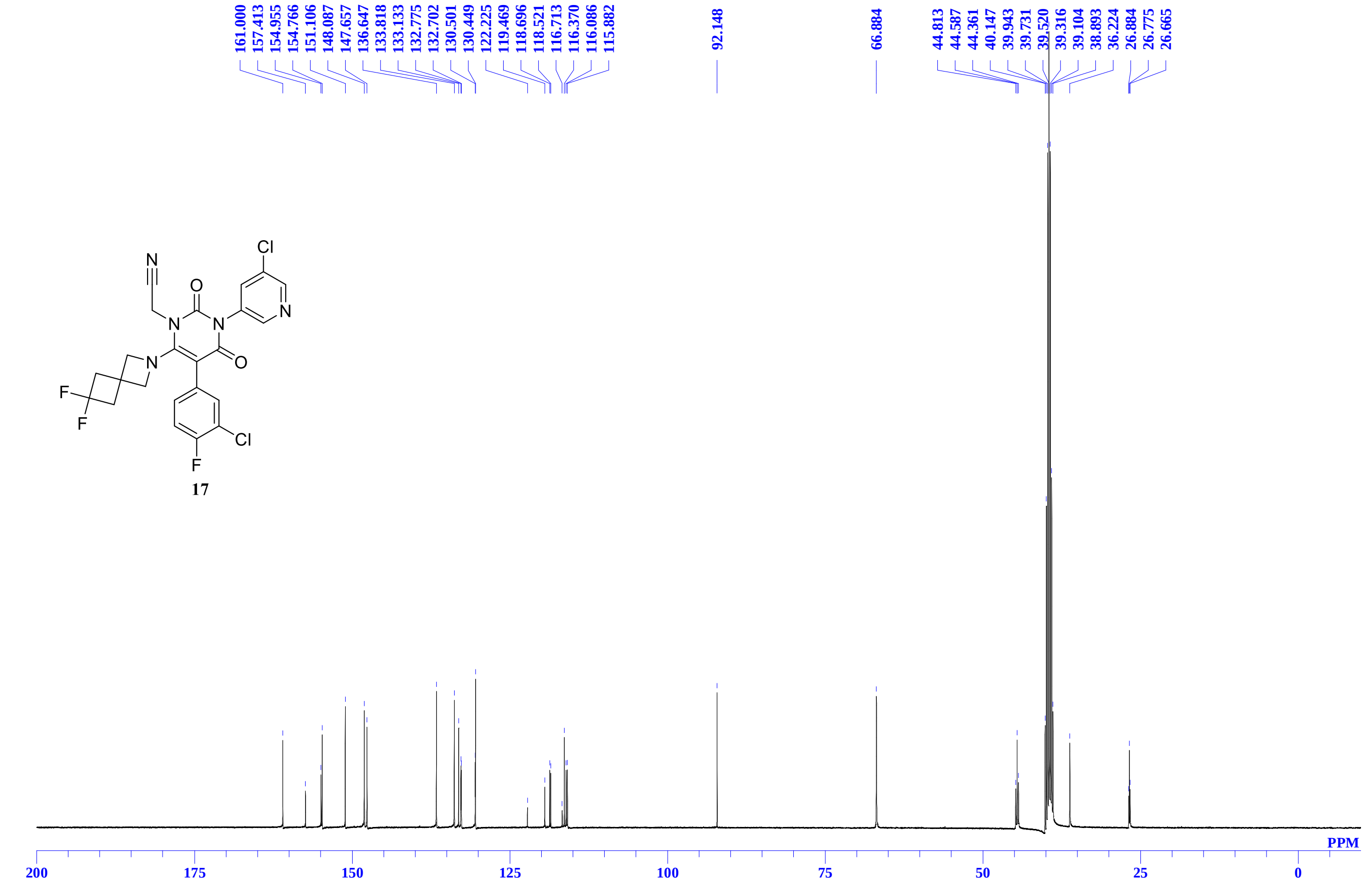

^{1}H NMR spectra of **18** in DMSO-d_6

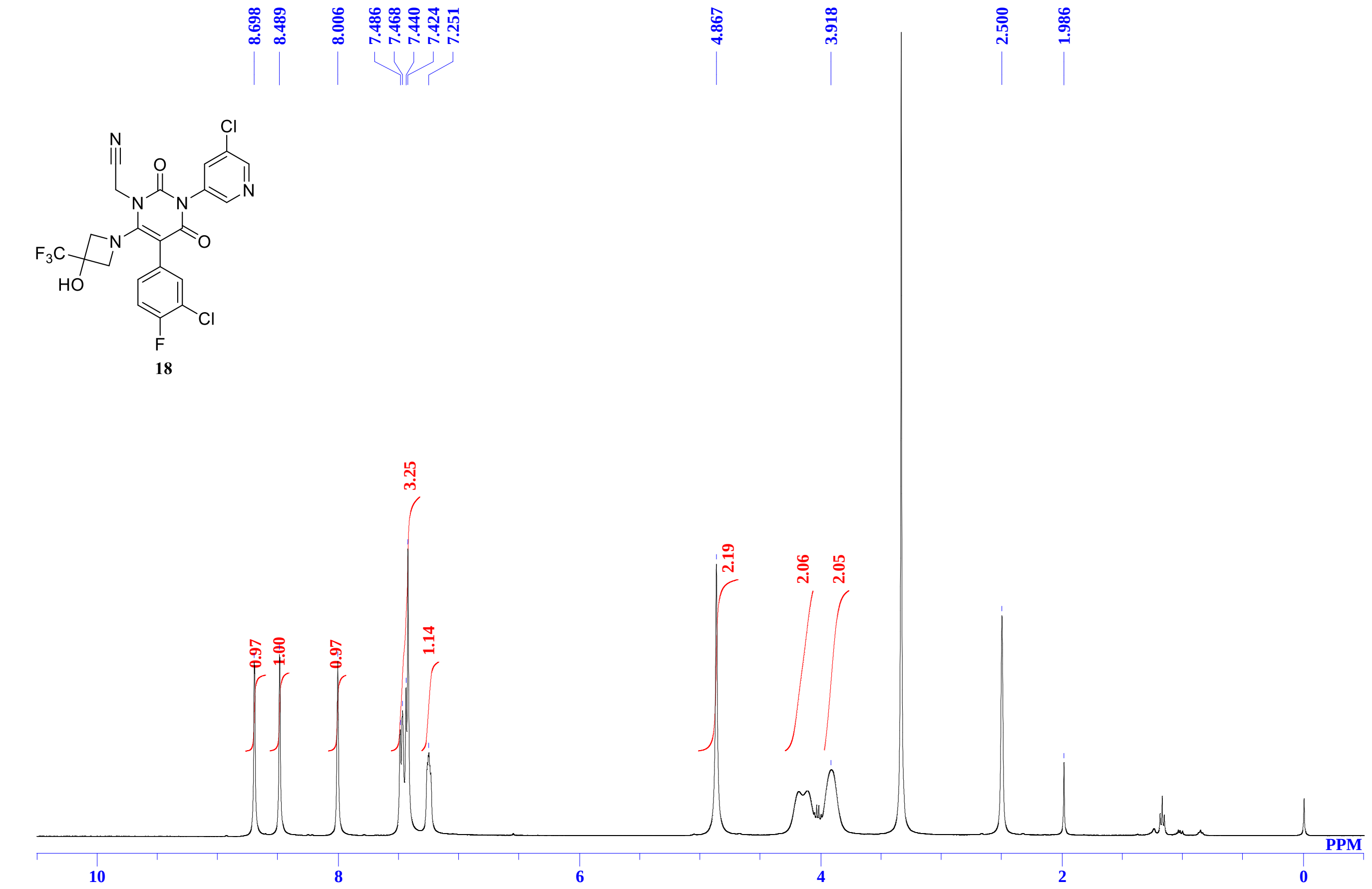

^{13}C NMR spectra of **18** in DMSO-d_6

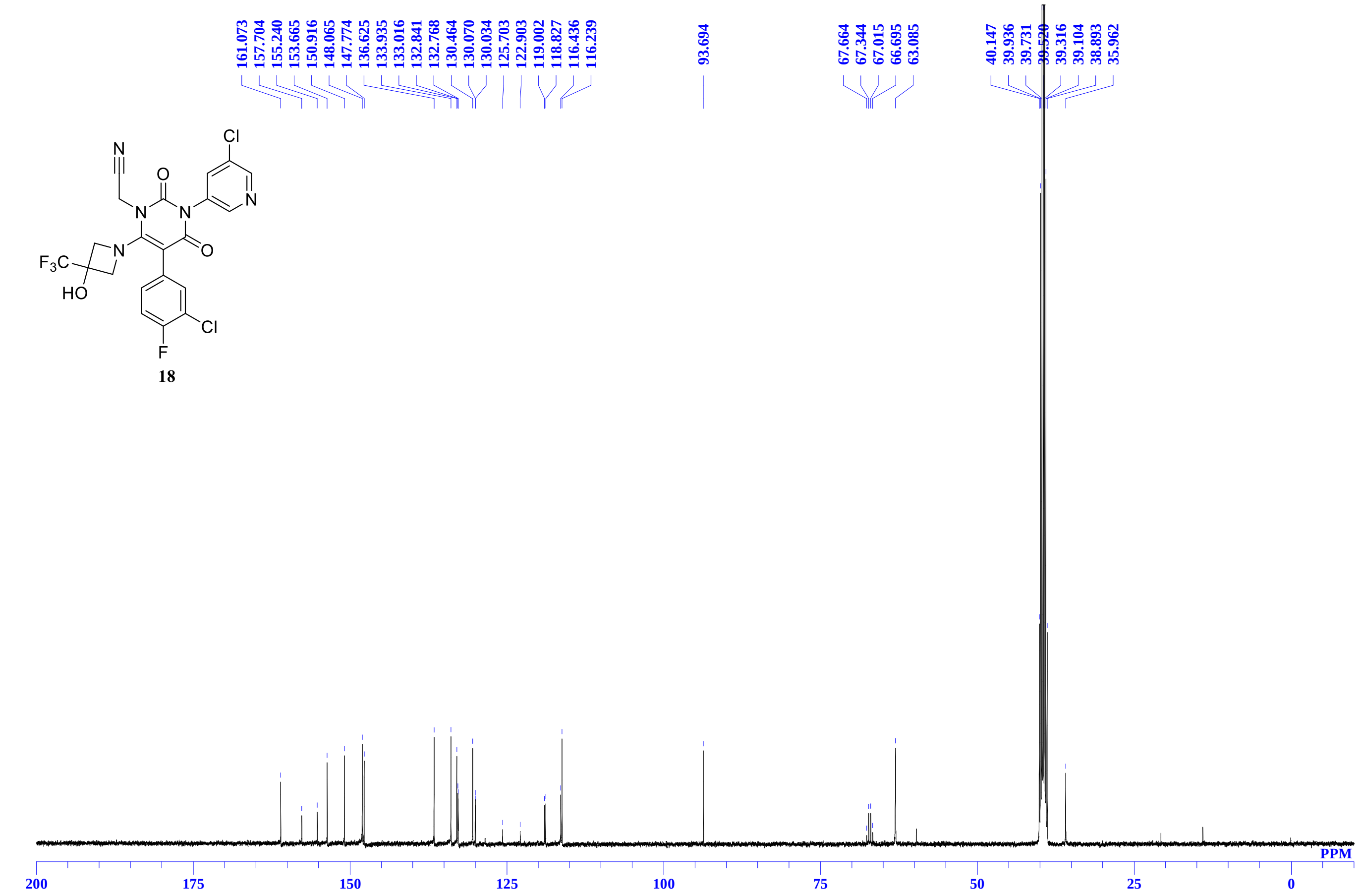

HPLC chromatogram of compound 17 (S-892216)

Analytical liquid chromatography of compound **17** (**S-892216**) was performed on a YMC-UltraHT Pro C18 Column (2.0 µm, 2.0 mm I.D.x100 mm, gradient from 10% to 90% B [A = 10 mmol/L $HCOONH_4$ / H_2O, B = 10 mmol/L $HCOONH_4$ / MeCN], flow rate: 0.4 mL/min) using a Shimadzu Nexera system equipped with LC-30AD binary gradient module and SPD-20AV detector (detection at 255 nm).

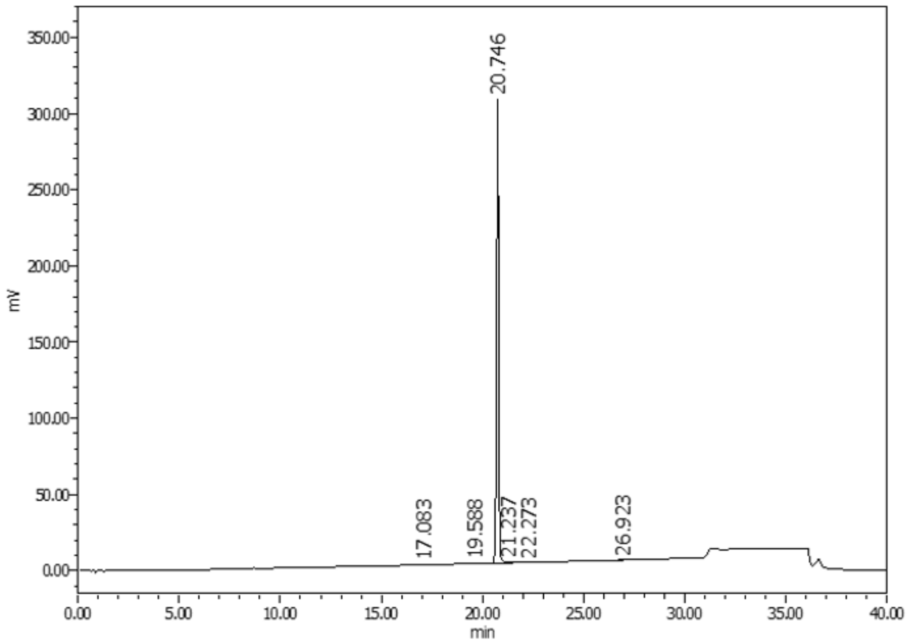

Time-program for gradient elution

Time (min)	Mobile phase B (%)
0	10
30	70
30.01	90
35	90
35.01	10
40	10

	Retention time (min)	Peak area (µV·sec)	Peak height (µV)	% area
1	17.1	1843	127	0.08
2	19.6	1616	79	0.07
3	20.7	2200909	304418	99.39
4	21.2	7832	820	0.35
5	22.3	959	54	0.04
6	26.9	1250	62	0.06

HPLC chromatogram of compound 18

Analytical liquid chromatography of compound **18** was performed on a Unison C18 Column (3.0 µm, 4.6 mm I.D.x75 mm, gradient from 10% to 90% B [A = 0.1% THF in H_2O, B = MeCN], flow rate: 1.0 mL/min, Method : 0-15min [B]=10-90%, 15-20min [B]=90%) using a Shimadzu Prominence system equipped with LC-20AD binary gradient module and SPD-20AV detector (detection at 254 nm)

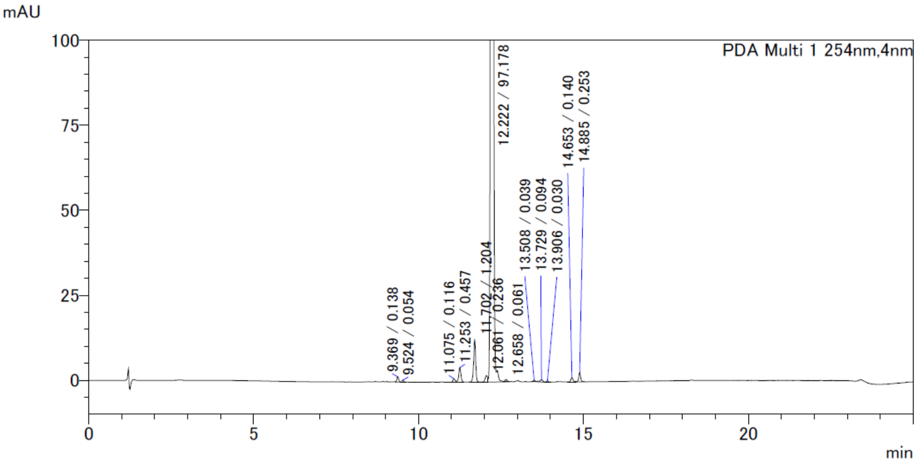

	Retention time (min)	Peak area (µ·sec)	Peak height (µV)	% area
1	9.369	6118	1610	0.138
2	9.524	2379	619	0.054
3	11.075	5167	1062	0.116
4	11.253	20259	4374	0.457
5	11.702	53425	12660	1.204
6	12.061	10481	1938	0.236
7	12.222	4310762	1100924	97.178
8	12.658	2702	568	0.061
9	13.508	1733	462	0.039
10	13.729	4191	674	0.094
11	13.906	1315	337	0.03
12	14.653	6196	1242	0.14
13	14.885	11223	2737	0.253

Physicochemical Properties of 17 (S-892216)

X-ray Powder Diffraction（XRPD）

A Crystal form of **S-892216** was analyzed by X-ray powder diffraction (XRPD) using SmartLab (Rigaku Corporation, Tokyo, Japan). The samples were placed in each hole (diameter = 2 mm, depth = 0.1 mm) on an aluminum plate, and their surfaces were smoothed by using a spatula. X-ray diffraction of the samples was performed with a 9 kW rotating anode using Cu Kα radiation (λ = 0.154186 nm) and a HyPix-3000 detector. The distance between the sample and the detector was 331 mm, and the diffractometer was equipped with a cross-beam optic. A parallel-slit collimator with 2.5° and a slit of 0.05 mm height and 0.5 mm width was used. The scan was performed from 3° to 32° (2θ) with β axis rotation (20 rpm) during data collection; the sampling step was 0.02° (2θ), and the count time was 40 s. The data were analyzed using Smart Lab Studio II X64 version 4.2.111.0 (Rigaku Corporation, Tokyo, Japan).

Thermogravimetry–Differential Thermal Analysis（TG-DTA）

Simultaneous measurements of the changes in weight and thermal profile as a function of temperature were performed by thermogravimetry–differential thermal analysis (TG–DTA) using a STA7200RV instrument (Hitachi High-Tech Science Corporation, Tokyo, Japan). The sample was placed into an aluminum pan and measured at 10 °C/min up to 350 °C. The results were analyzed using TA7000 standard analysis, version 11.2 (Hitachi High-Tech Science Corporation, Tokyo, Japan).

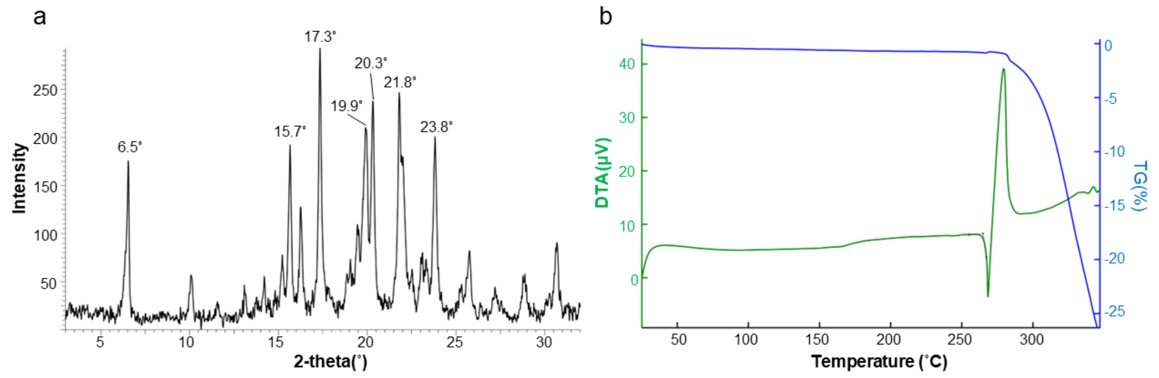

Figure S2. Physicochemical Properties of **S-892216**. (a) XRPD patterns. (b) TG-DTA.

HRMS Spectra for compound 17 (S-892216)

The HRMS data were measured using the Thermo Fisher Scientific Q Exactive Focus with Heatead-ESI and a Shimadzu Nexera system (LC-30AD, CBM-20A, SIL-30ACAM, CTO-20AC, DGU-20A$_{5R}$, FCV-20AH$_2$). Elution conditions: column, YMC-Triat C18 Column, 2.1 × 30 mm, 2.5 μm particle size; column temperature, 30°C; solvent A, water (0.1% formic acid); solvent B, MeCN (0.1% formic acid); gradient: 5−95% B in 3.5 min, 100% B in 0.50 min, 5% B in 1.0 min, 5.0 min total run time; flow rate, 0.5 mL/min. LC/MS conditions, ESI in positive mode; temperature, 380°C; ion spray voltage, 3500 V.

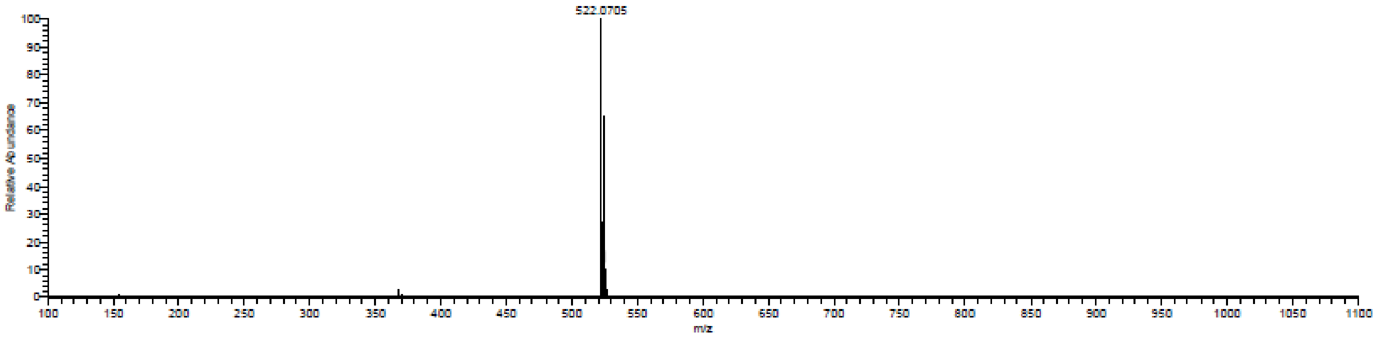

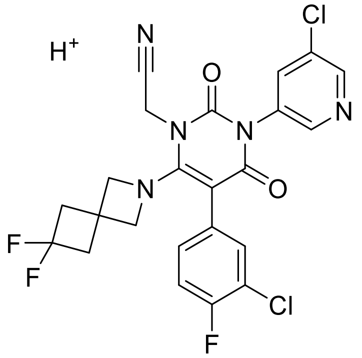

Chemical Formula: $C_{23}H_{17}Cl_2F_3N_5O_2^+$
Exact Mass: 522.0706

References

S1. Watari, R.; Tamura, N.; Yoshida, S.; Kido, Y.; Matsuzaki, T. Minimal Involvement of P-gp and BCRP in Oral Absorption of Ensitrelvir, An Oral SARS-CoV-2 3C-like Protease Inhibitor, in a Non-Clinical Investigation. *J. Pharm. Sci.*, **2024**, *113*, 2871– 2878, DOI: 10.1016/j.xphs.2024.06.009

S2. Unoh, Y.; Uehara, S.; Nakahara, K.; Nobori, H.; Yamatsu, Y.; Yamamoto, S.; Maruyama, Y.; Taoda, Y.; Kasamatsu, K.; Suto, T.; Kouki, K.; Nakahashi, A.; Kawashima, S.; Sanaki, T.; Toba, S.; Uemura, K.; Mizutare, T.; Ando, S.; Sasaki, M.; Orba, Y.; Sawa, H.; Sato, A.; Sato, T.; Kato, T.; Tachibana, Y. Discovery of S-217622, a Noncovalent Oral SARS-CoV-2 3CL Protease Inhibitor Clinical Candidate for Treating COVID-19. *J. Med. Chem.* **2022**, *65* (9), 6499– 6512, DOI: 10.1021/acs.jmedchem.2c00117

S3. Uehara, T.; Yotsuyanagi, H.; Ohmagari, N.; Doi, Y.; Yamato, M.; Imamura, T.; Sakaguchi, H.; Fukushi, A.; Takeda, Y.; Baba, K.; Nobori, H.; Miyamoto, T.; Arita, S.; Dodo, R.; Shimba, A.; Fukao, K.; Shishido, T.; Tsuge, Y.; Mukae, H. Ensitrelvir treatment-emergent amino acid substitutions in SARS-CoV-2 3CLpro detected in the SCORPIO-SR phase 3 trial, *Antiviral Res.* **2025**, *236*, 106097, DOI: 10.1016/j.antiviral.2025.106097

S4. Kuroda, T.; Nobori, H.; Fukao, K.; Baba, K.; Matsumoto, K.; Yoshida, S.; Tanaka, Y.; Watari, R.; Oka, R.; Kasai, Y.; Inoue, K.; Kawashima, S.; Shimba, A.; Hayasaki-Kajiwara, Y.; Tanimura, M.; Zhang, Q.; Tachibana, Y.; Kato, T.; Shishido, T. Efficacy comparison of 3CL protease inhibitors ensitrelvir and nirmatrelvir against SARS-CoV-2 in vitro and in vivo. *J. Antimicrob. Chemother.* **2023**, 78 (4), 946– 952, DOI: 10.1093/jac/dkad027

S5. Uemura, K.; Nobori, H.; Sato, A.; Toba, S.; Kusakabe, S.; Sasaki, M.; Tabata, K.; Matsuno, K.; Maeda, N.; Ito, S.; Tanaka, M.; Anraku, Y.; Kita, S.; Ishii, M.; Kanamitsu, K.; Orba, Y.; Matsuura, Y.; Hall, WW.; Sawa, H.; Kida, H.; Matsuda, A.; Maenaka, K. 2-thiouridine is a broad-spectrum antiviral nucleoside analogue against positive-strand RNA viruses. *Proc. Natl. Acad. Sci. USA.* **2023**, 120 (42), e2304139120, DOI: 10.1073/pnas.2304139120. Epub 2023

S6. Sahara, N.; Hirano, Y.; Thien, P. L.; Yamakawa, K.; Ohashi, E.; Shibuya, S.; Komano, K.; Hirai, K.; Masui, M.; Kato, G.; Higuchi, T.; Shimazaki, Y.; Kawajiri, T.; Oohara, T.; Tsuno, N.; Ohara, T. Development of a Manufacturing Process for S-892216 Part I: A Novel Method for Constructing a Multi-Substituted Barbiturate Skeleton for Scalable Synthesis. *ChemRxiv.* **2025**, DOI:10.26434/chemrxiv-2025-sdmw6 This content is a preprint and has not been peer-reviewed.

S7. Le, T. P.; Kawajiri, T.; Sahara, N.; Kato, G.; Hosoya, M.; Oohara, T.; Agura, K.; Ohara, T.; Goda, S. Development of a Manufacturing Process for S-892216 Part II: Improvements Toward Commercially Feasible Process. *ChemRxiv.* **2025**, DOI:10.26434/chemrxiv-2025-0wdzt This content is a preprint and has not been peer-reviewed.
